# Distinct Viral and Mutational Spectrum of Endemic Burkitt Lymphoma

**DOI:** 10.1371/journal.ppat.1005158

**Published:** 2015-10-15

**Authors:** Francesco Abate, Maria Raffaella Ambrosio, Lucia Mundo, Maria Antonella Laginestra, Fabio Fuligni, Maura Rossi, Sakellarios Zairis, Sara Gazaneo, Giulia De Falco, Stefano Lazzi, Cristiana Bellan, Bruno Jim Rocca, Teresa Amato, Elena Marasco, Maryam Etebari, Martin Ogwang, Valeria Calbi, Isaac Ndede, Kirtika Patel, David Chumba, Pier Paolo Piccaluga, Stefano Pileri, Lorenzo Leoncini, Raul Rabadan

**Affiliations:** 1 Department of Systems Biology, Columbia University College of Physicians and Surgeons, New York, New York, United States of America; 2 Department of Biomedical Informatics, Columbia University College of Physicians and Surgeons, New York, New York, United States of America; 3 Department of Medical Biotechnologies, Section of Pathology, University of Siena, Siena, Italy; 4 Department of Experimental, Diagnostic, and Specialty Medicine (DIMES), S. Orsola-Malpighi Hospital, Bologna University School of Medicine, Bologna, Italy; 5 School of Biological and Chemical Sciences, Queen Mary University of London, London, United Kingdom; 6 Lacor Hospital, Gulu, Uganda; 7 Moi University, Eldoret, Kenya; 8 Unit of Haematopathology, European Institute of Oncology, Milan and Bologna University School of Medicine, Bologna, Italy; Wistar Institute, UNITED STATES

## Abstract

Endemic Burkitt lymphoma (eBL) is primarily found in children in equatorial regions and represents the first historical example of a virus-associated human malignancy. Although Epstein-Barr virus (EBV) infection and *MYC* translocations are hallmarks of the disease, it is unclear whether other factors may contribute to its development. We performed RNA-Seq on 20 eBL cases from Uganda and showed that the mutational and viral landscape of eBL is more complex than previously reported. First, we found the presence of other herpesviridae family members in 8 cases (40%), in particular human herpesvirus 5 and human herpesvirus 8 and confirmed their presence by immunohistochemistry in the adjacent non-neoplastic tissue. Second, we identified a distinct latency program in EBV involving lytic genes in association with *TCF3* activity. Third, by comparing the eBL mutational landscape with published data on sporadic Burkitt lymphoma (sBL), we detected lower frequencies of mutations in *MYC*, *ID3*, *TCF3* and *TP53*, and a higher frequency of mutation in *ARID1A* in eBL samples. Recurrent mutations in two genes not previously associated with eBL were identified in 20% of tumors: *RHOA* and *cyclin F* (*CCNF*). We also observed that polyviral samples showed lower numbers of somatic mutations in common altered genes in comparison to sBL specimens, suggesting dual mechanisms of transformation, mutation versus virus driven in sBL and eBL respectively.

## Author Summary

Burkitt lymphoma is endemic in sub-Saharan Africa and affects primarily children of age 4–7 years. Historically, it was one of the first tumors associated with a virus (EBV) and bearing a translocation involving an oncogene, i.e. *MYC*. There are three distinct clinical variants of Burkitt lymphoma according to the World Health Organization: sporadic, endemic and immunodeficiency-related. Although there has been some recent work on the molecular characterization of sporadic Burkitt lymphomas, little is known about the pathogenesis of endemic cases. In this work, we analyzed 20 samples of RNASeq from Burkitt lymphoma collected in Lacor Hospital (Uganda, Africa) and validated in an extension panel of 73 samples from Uganda and Kenya. We identify the presence in the adjacent non-neoplastic tissue of other herpesviridae family members in 53% of the cases, namely cytomegalovirus (CMV) and Kaposi sarcoma herpesvirus (KSHV). We also demonstrate expression of EBV lytic genes in primary tumor samples and find an inverse association between EBV lytic expression and *TCF3* activity. When studying the mutational profile of endemic Burkitt tumors, we find recurrent alterations in genes rarely mutated in sporadic Burkitt lymphomas, i.e. *ARID1A*, *CCNF* and *RHOA*, and lower numbers of mutations in genes previously reported to be commonly mutated in sporadic cases, i.e. *MYC*, *ID3*, *TCF3*, *TP53*. Together, these results illustrate a distinct genetic and viral profile of endemic Burkitt lymphoma, suggesting a dual mechanism of transformation (mutation versus virus driven in sBL and eBL respectively).

## Introduction

Burkitt lymphoma (BL) is the first human cancer to be associated with the Epstein-Barr virus (EBV), the first tumor to exhibit a chromosomal translocation activating an oncogene (*MYC*), and the first lymphoma to be associated with human immunodeficiency virus (HIV) infection. The World Health Organization[1] classification describes three clinical variants of BL: endemic, sporadic, and immunodeficiency-related. These variants are similar in morphology, immunophenotype, and genetics. While the sporadic variant (sBL) occurs outside of Africa and is rarely associated with EBV infection, the endemic variant (eBL) arises mainly in Africa and is associated with malaria endemicity and EBV infection in almost all cases. Epidemiological studies have shown that malaria and EBV combined do not fully explain the distribution of eBL in high risk regions[2]. Malaria and EBV are in fact ubiquitous within the lymphoma belt of Africa, suggesting that other etiologic agents may be involved[3]. However, it is unclear what other epidemiological factors could play a role in the genesis of eBLs.

Three types of EBV latency have been described in EBV-related lymphomas according to the pattern of EBV nuclear antigen (EBNA) and the latent membrane protein (LMP) expression, namely latency I, II, and III[4]. Specifically, latency I is usually associated with eBL and it denotes a transcriptional program in which an EBV infection does not produce virions and expresses a single protein, EBNA-1. While the latency I program has been extensively characterized *in vitro*, a different form of latency has been recently reported in 15% of eBL that uses a different set of promoters. Termed Wp-restricted latency[5], this program shows a homogeneous host expression signature[6] characterized by down-regulation of *BCL-6* and up-regulation of *IRF-4* and *BLIMP-1*. Other reports have described latency program heterogeneity at single cell level[7] and low expression of *LMP* genes in a fraction of cases[8,9]. Heterogeneous EBV transcription profiles with LMP expression have been recently reported in some cases of AIDS-related and sporadic BL[10], but extensive data on endemic cases are not available yet. These studies indicate that the transcriptional EBV programs of primary eBL could be more complex than expected across cases and within individuals. Therefore, the exact role of EBV has remained elusive and further investigation is required.

The genetic hallmark of all three clinical variants of BL is the t(8;14) translocation involving the juxtaposition of the immunoglobulin heavy chain locus (IGH) with the *MYC* oncogene[11]. However, although transgenic mice expressing *MYC* under the control of the intronic *IGH* enhancer (Eμ) develop B cell lymphomas[12], successive molecular characterization demonstrated that this model does not fully recapitulate the human disease. The comparison between the gene expression profile (GEP) of BL and diffuse large B-cell lymphoma (DLBCL) highlighted a distinct signature of BL characterized by the expression of both *MYC* targets and germinal-center B-cell genes[13]. Furthermore, hypermutation and different breakpoint patterns of *IGH/MYC* translocation[14,15] suggests that the origin of human BL derives from aberrant class switching in the germinal center (GC), while transgenic *IGH/MYC* mice typically arise from precursor/naive B-cells. The more accurate *PI3K/MYC* transgenic mouse model by Sander *et al[16]* better recapitulates the human phenotype of BL and highlights the importance of the PI3K pathway in the disease. Moreover, GEP analysis has demonstrated that the transcriptional profile of eBL is different from that observed in sBL[17]. Recent studies have unveiled the genetic landscape of sBL characterized by mutations affecting the B-cell receptor (BCR) pathway and in particular the transcription factor *TCF3*, its negative regulator *ID3*, the cell-cycle G1/S regulator *CCND3*[18,19], and the chromatin-remodeling gene *ARID1A*[20]. On the contrary, very little is known about the spectrum of alterations in eBL, how it might differ from that of sBL, the correlations between host mutation and viral infection, and the specific viral/host transcriptional programs.

In this study, we aim to characterize the presence of other potential agents, to define the EBV transcriptional profile and to link these profiles to the mutational status of new and previously reported genes. We provide a characterization of the mutational and viral landscape of eBL using 20 cases from Uganda. RNA-Seq, in combination with targeted sequencing technology on a larger cohort of cases, allows the identification, validation and assessment of the recurrence of new somatic mutations. In addition, in contrast with earlier microarray-based expression studies, RNA-Seq provides the opportunity to identify and associate microbial and tumor mutational and expression profiles.

## Results

### Endemic BL is associated with multiple viral infections

**Fig 1:**
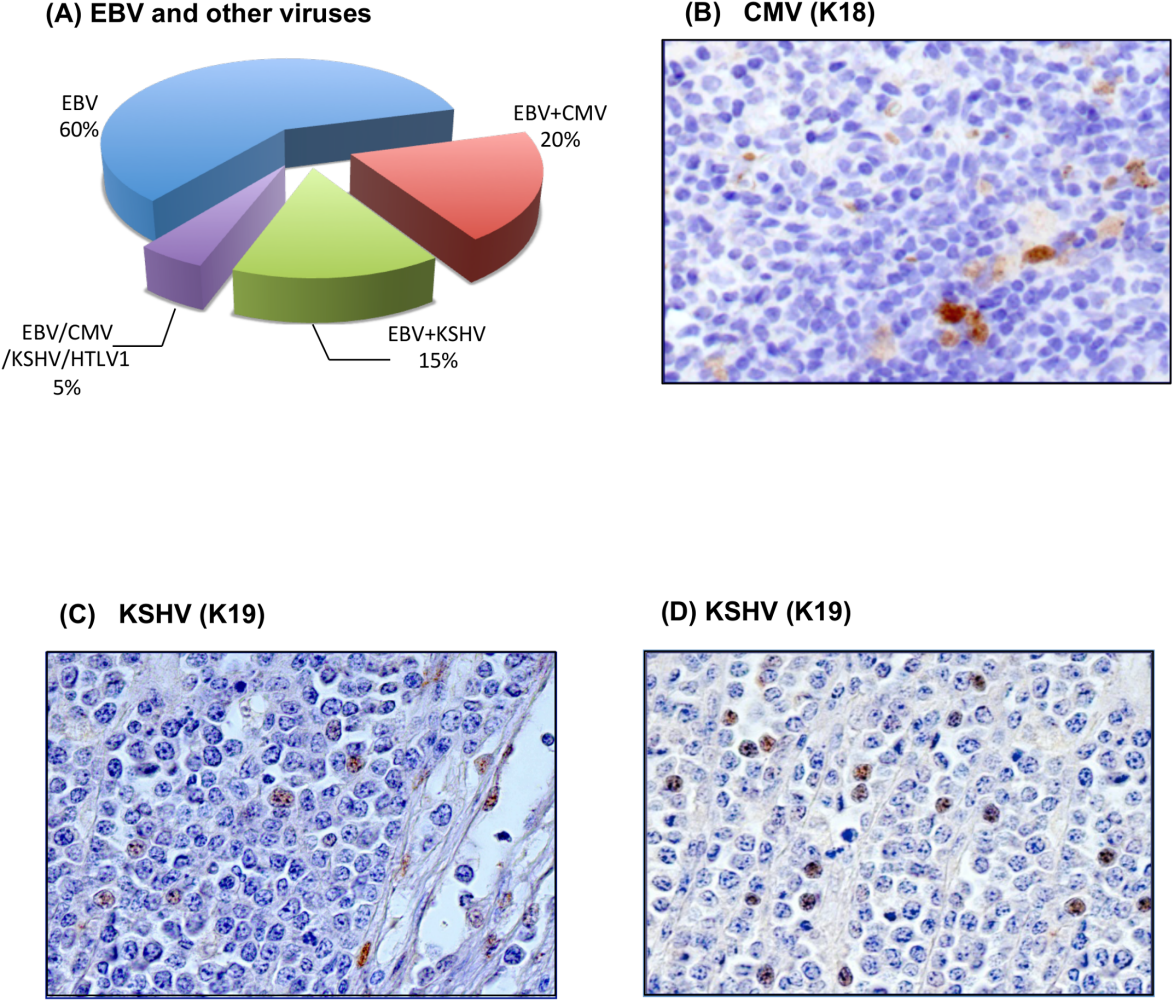
(A) RNA-Seq technology reveals the presence of EBV and of other viruses. In particular, 5/20 cases contain human herpesvirus 5 (CMV), 4/20 human herpesvirus 8 (KSHV), and 1/20 human T-lymphotropic virus 1 (HTLV-1). (B) Immunohistochemical evaluation demonstrates the presence of CMV in the stromal cells in the adjacent reactive lymphoid tissue. CMV stain, Original Magnification (O.M.): 40x. (C) KSHV positivity is shown, respectively in few neoplastic cells and in the endothelial cells within the neoplastic proliferation. LANA-1 (LN53 antibody), O.M.: 40x; (D) LANA-1 (AT4C11 antibody) O.M.: 40x.

To identify new pathogens in eBL, we applied Pandora, a new pipeline for the characterization of tumor microbiomes, to a discovery cohort of 20 RNA-Seq samples. We established a read cutoff on the basis of those samples that tested positive for RNA *in situ* hybridization (ISH) of the EBER transcript. Since ISH validated all the RNA-Seq samples as positive, we established the threshold to call a virus present in a particular sample as the minimal number of reads detecting EBV (S1 Fig). Next, we established the EBV subtype by aligning RNA-Seq reads to the genomes of both EBV type I and type II and deduced type I as the closest genotype. In addition to EBV, RNA-Seq revealed the presence of other viruses. In particular, 5/20 cases contained human herpesvirus 5 (HHV5, cytomegalovirus, CMV), 4/20 human herpesvirus 8 (HHV8, Kaposi sarcoma herpes virus, KSHV), and 1/20 human T-lymphotropic virus 1 (HTLV-1) (Fig 1A, S2 Fig and S3 Fig). Human immunodeficiency virus (HIV) was not detected in any case, confirming that pediatric eBL is rarely associated with the immunodeficiency syndrome[21]. Nested PCR and immunohistochemical (IHC) analysis performed on all 20 original samples confirmed the presence of all the viruses in the discovery cohort (S1A Table). To assess whether RNA-Seq findings generalize for EBV, CMV, KSHV, and HTLV-1, we assayed for the presence of these four viruses in 20 additional cases from western Kenya by IHC (S1B Table). In this Kenyan cohort, EBV was detected in 20/20 samples, CMV in 8/20 samples (Fig 1B and S4 Fig), KSHV in 7/20 samples (Fig 1C and 1D, and S5 Fig), and HTLV-1 in 0/20 samples. Therefore, over the 40 cases, we report the overall viral infection frequencies of 40/40 (100%) for EBV, 13/40 (32.5%) for CMV, 11/40 (27.5%) for KSHV, and 1/40 (2.5%) for HTLV-1. IHC analysis demonstrated the presence of CMV in the stromal cells and macrophages localized within the tumors and in the adjacent reactive lymphoid tissue (Fig 1B, S2 Fig and S4 Fig). KSHV was identified not only in normal B-lymphocytes and endothelial cells from the adjacent reactive lymphoid tissue (S3 Fig and S5 Fig), but also in one case in about 5–10% of neoplastic cells (Fig 1C and 1D). HTLV-1 was detected in reactive T-lymphocytes in the only positive case of the discovery cohort. Sections of the samples incubated with the secondary antibody alone and sections of reactive lymphoid tissue were used as negative controls. Sections of lymph nodes with infectious mononucleosis were used as positive control for EBV. Next, we compared the viral landscape of endemic and sporadic cases by analyzing 27 RNA-Seq sBL samples from Schmitz *et al*.[19] with Pandora. The analysis showed the presence of EBV and HIV respectively in 4/27 (15%) and in 1/27 (4%) cases, consistent with several literature sources[22].

### Epstein-Barr virus latent and lytic infection in BL

**Fig 2:**
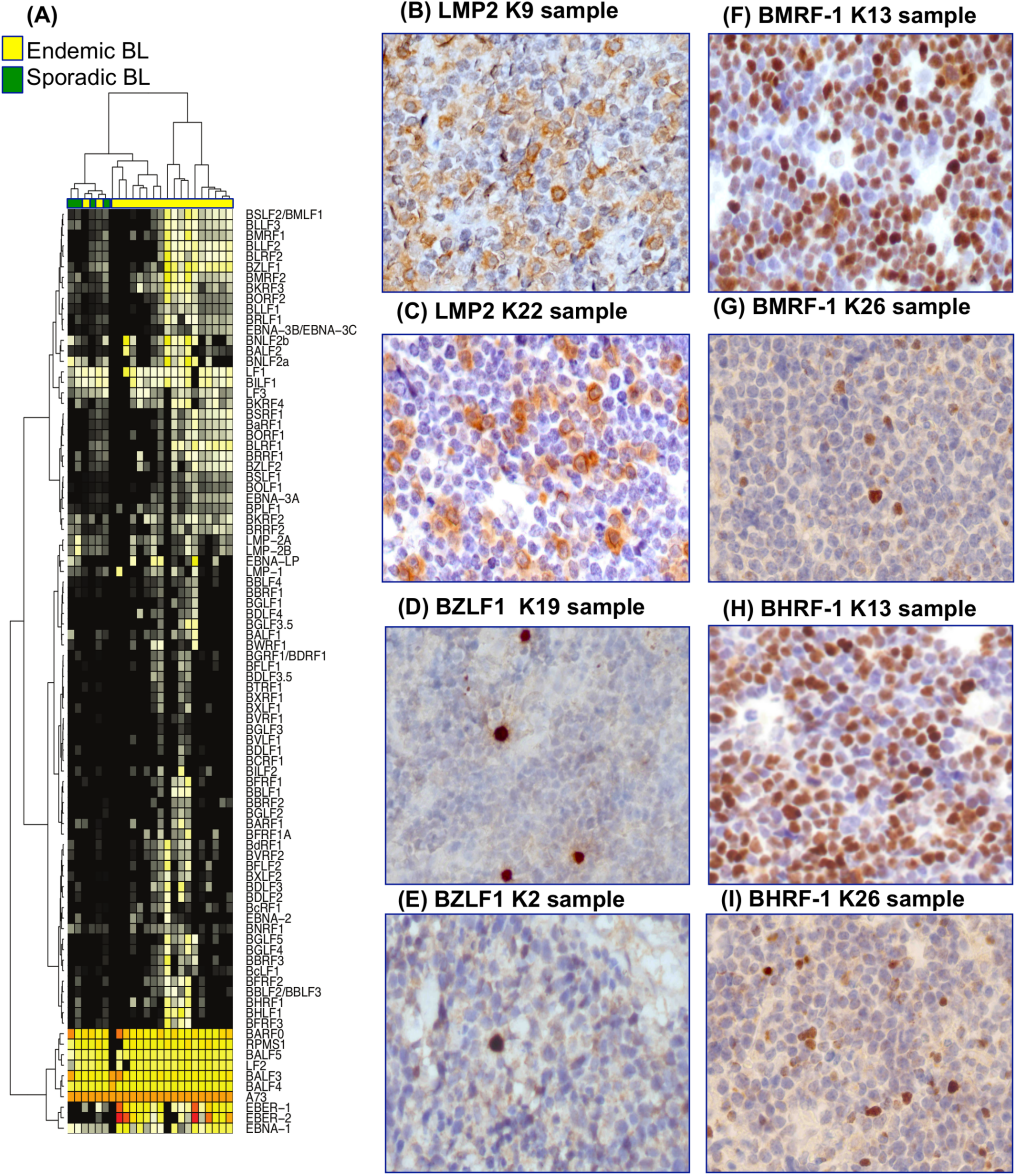
(A) Unsupervised hierarchical clustering of expressed EBV genes demonstrates a diversity of non-canonical latency-associated gene expression programs with a subset of viral episome initiating lytic reactivation as indicated by expression of genes corresponding to the lytic program. (B) LMP-2A is expressed by 40 to 50% of neoplastic cells. LMP-2A stain, O.M.: 40x; (C) LMP-2A expression is identified in a proportion of neoplastic cells ranging from 20 to 30%. LMP-2A stain, O.M.: 40x; (D) BZLF1/ZEBRA positivity is expressed by 5 to 10% of neoplastic cells. BZLF1/ZEBRA stain, O.M.: 40x; (E) BZLF1/ZEBRA expression is detected in few neoplastic cells. BZLF1/ZEBRA stain, O.M.: 40x; (F) BMRF-1/Ea-D expression is observed in 50% of neoplastic cells. BMRF-1/Ea-D stain, O.M.: 40x; (G) BMRF-1/Ea-D protein expression in 5% to 10% of neoplastic cells is shown. BMRF-1/Ea-D stain, O.M.: 40x; (H) BHRF-1/Ea-R staining is found in 60% of neoplastic cells. BHRF-1/Ea-R stain, O.M.: 40x; (I) BHRF-1/Ea-R is expressed in 10% of neoplastic cells. BHRF-1/Ea-R stain, O.M.: 40x.

Beyond identification of EBV presence, RNA-Seq enabled us to quantitatively analyze the viral transcriptional program. In addition to EBER-1 and EBER-2 transcripts, expression analysis of the viral genes showed the expression of *EBNA-1*, a gene associated to latency I type, in 18/20 cases (Fig 2A). We also detected either *LMP-1* or *LMP-2A*, characterizing the latency II type, in 13/20 samples (65%), and also EBNA-2 in 1/20 cases (5%). Interestingly, 2/20 cases (10%) were characterized by the expression of *EBNA-3A/B/C/LP*, together with the lytic gene *BHRF-1*, suggesting a Wp-restricted program[23]. However, the specific analysis of EBV isoforms showed the presence of H2-HF splicing event, which is hallmark of lytic *BHRF-1* expression[24–26](S6 Fig). Unsupervised hierarchical clustering of expressed EBV genes demonstrated two main clusters distinguished largely by gene products involved in EBV replication (BALF-2, BCRF-1, BHRF-1, BILF-1, BMRF-1, BNLF-2a, BZLF-1). The expression of these genes suggests a non-canonical latency program of the virus with a subset of viral episomes initiating lytic reactivation[23].

Due to the heterogeneity of the viral transcriptional programs, we aimed to validate the latency type by performing RT-qPCR for the *EBNA-1*, *LMP-1*, *LMP-2A*, *EBNA-2*, *EBNA-3C*, and *BHRF-1* transcripts across an additional series of 26 cases from an extended cohort of samples from Kenya. *EBNA-1* was detected in 26/26 (100%), *LMP-1* and *LMP-2* in respectively 5/26 (20%) and 20/26 (75%) cases (S3 Table and S7A Fig), *EBNA-2* in 0/26 (0%), and the combination of *EBNA-3C* and *BHRF-1* in 4/26 (15%). These results are largely consistent with the RNA-Seq data with the exception of *LMP-1* that has been detected at higher frequency in RNA-Seq (S2 Table). Next, we evaluated the lytic cycle activation and found *BILF-1*, *BALF-4*, and *LF-2* in all 26 cases, whereas we observed the expression of *BALF-2* in 23/26 (90%), *BHRF-1* in 20/26 (80%), *BZLF-1* and *BMRF-1* in 15/26 (60%), *BNLF-2a* in 13/26 (50%), and *BCRF-1* in 11/26 (45%) of the cases (S3 Table and S7B–S7D Fig).

We then validated the expression of all the available encoded-proteins by IHC using stringent positive and negative controls as reported in Materials and Methods. Overall, IHC evaluation confirmed a non-canonical latency associated program with the expression of some proteins characterizing latency II (i.e. LMP-1 in 2/26 and LMP-2A in 17/26 of the cases); however, there was heterogeneity in the intensity of protein staining and in the proportion of positive tumor cells. LMP-1 was detected in few cells, whereas LMP-2A was identified in a proportion of cells ranging from 25% to 50% (Fig 2B and 2C). EBV replication was assessed by nuclear expression of the immediate-early BZLF-1/ZEBRA and early BMRF-1/Ea-D, BHRF-1/Ea-R lytic proteins (Fig 2D–2I). There was positive staining in the neoplastic cells for BZLF1, BHRF-1/Ea-R and BMRF-1/Ea-D, respectively in 11/26 (40%), 16/26 (60%), and 13/26 (50%) of the cases (S3 Table).

Finally, we compared the patterns of latent and lytic gene expression between endemic and sporadic BLs using the 4 EBV-positive sBLs of the 27 RNA-Seq samples from Schmitz *et al*.^16^ We observed the expression of *BHRF-1* and *BMRF-1* in 1 case; *BZLF-1* was present in 2 cases and *LMP-2A* in 4 cases.

### Mutational landscape of eBL and correlation with EBV presence

**Fig 3:**
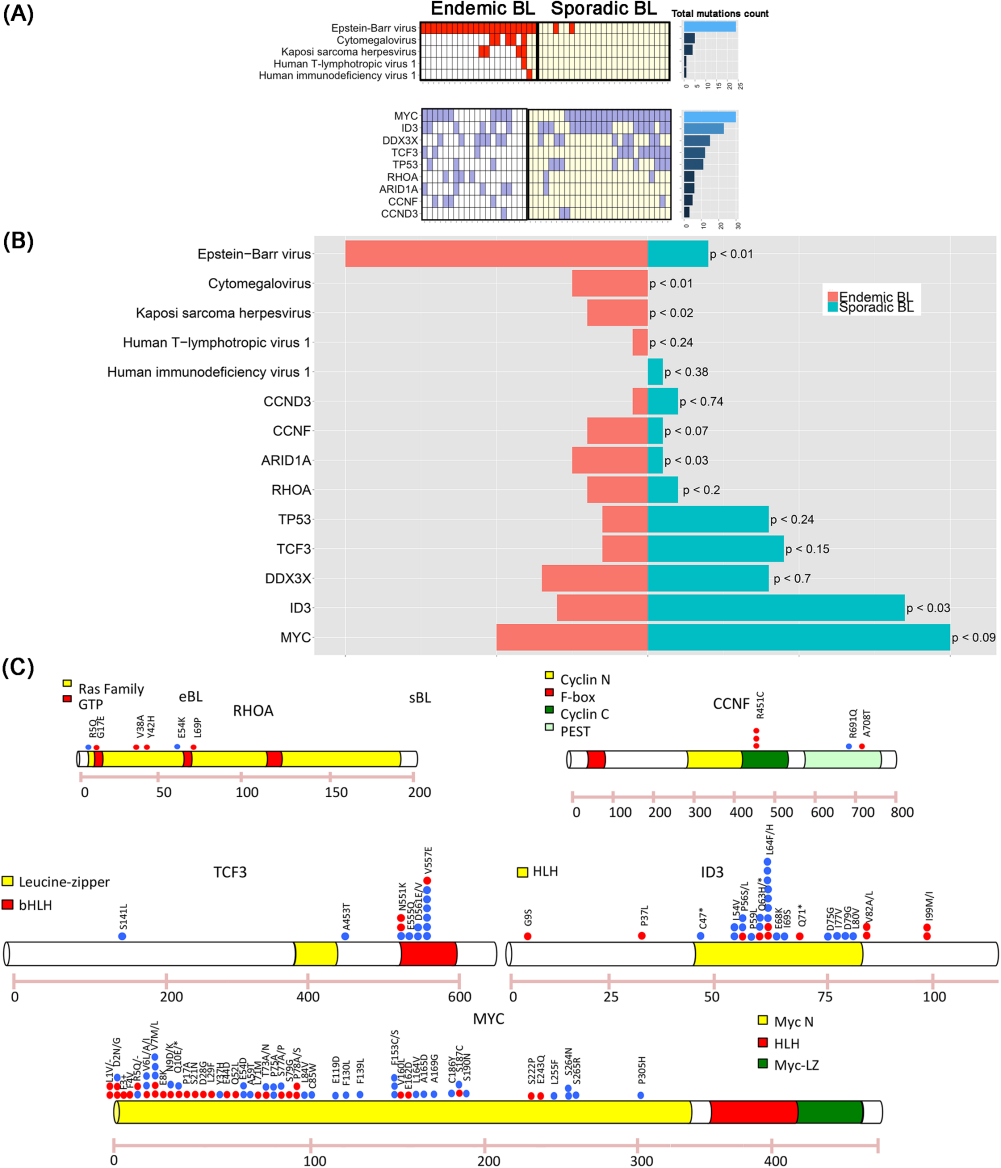
(A) The presence of mutations in genes previously described in BL is reported, including *MYC* (50%), *DDX3X* (35%), *ID3* (30%), *ARID1A* (25%), *RHOA* (20%), *TCF3* and *TP53* (15%), and *CCND3* 1/20 (5%). In addition, a new mutation is shown, involving *CCNF* and detected in 20% of the cases. (B) Bar plot showing the frequency comparison of virus presence and driver mutations between endemic and sporadic BL. For each comparison we report the p-value associated with rejecting the null hypothesis of equal eBL and sBL prevalences. (C) Distribution of mutations in 5 driver genes. Red points indicate endemic BL, while blue points the sporadic ones.

To identify the genes that are somatically mutated in eBL, we applied the SAVI algorithm[27] to the cohort of 20 RNA-Seq samples (see Material and Methods for gene selection criteria). Our analysis identified 13 genes recurrently mutated in more than 4 samples. We confirmed the presence of mutations in genes previously reported in BL literature[18,19,28] (Fig 3A–3C and S4 Table), including *MYC* in 10/20 (50%), *DDX3X* in 7/20 (35%), *ID3* in 6/20 (30%), *ARID1A* in 5/20 (25%), *RHOA* in 4/20 (20%), *TCF3* and *TP53* in 3/20 (15%), *CCND3* in 1/20 (5%) of the cases. In addition, we found recurrent mutations in one gene not reported so far: *CCNF*, detected in 4 out of the 20 cases (20%). Since *RHOA* mutations have not been previously detected in eBL and *CCNF* mutation was a new discovery, their prevalence as specific mutations was further assessed using Sequenom technology on an extended panel of 66 neoplastic samples *plus* 7 cases with matched normal controls (S8A and S8B Fig). Recurrent mutations in *RHOA* were found in 6/73 eBL cases (8%), and in 0/7 normal samples. Two of the 6 *RHOA* mutations occurred in paired eBL/normal cases, confirming that the alterations are somatic (S5 Table). Recurrent mutations in codon 451 of *CCNF* were found in 14/73 eBL cases (19%), and in 0/7 normal samples. One of the 14 *CCNF* mutations occurred in a paired eBL/normal case, showing that also *CCNF* alteration is somatic. Direct sequencing of genomic DNA confirmed all the mutations identified by Sequenom tecnnology and RNAseq (S9 Fig and S10 Fig).

**Fig 4:**
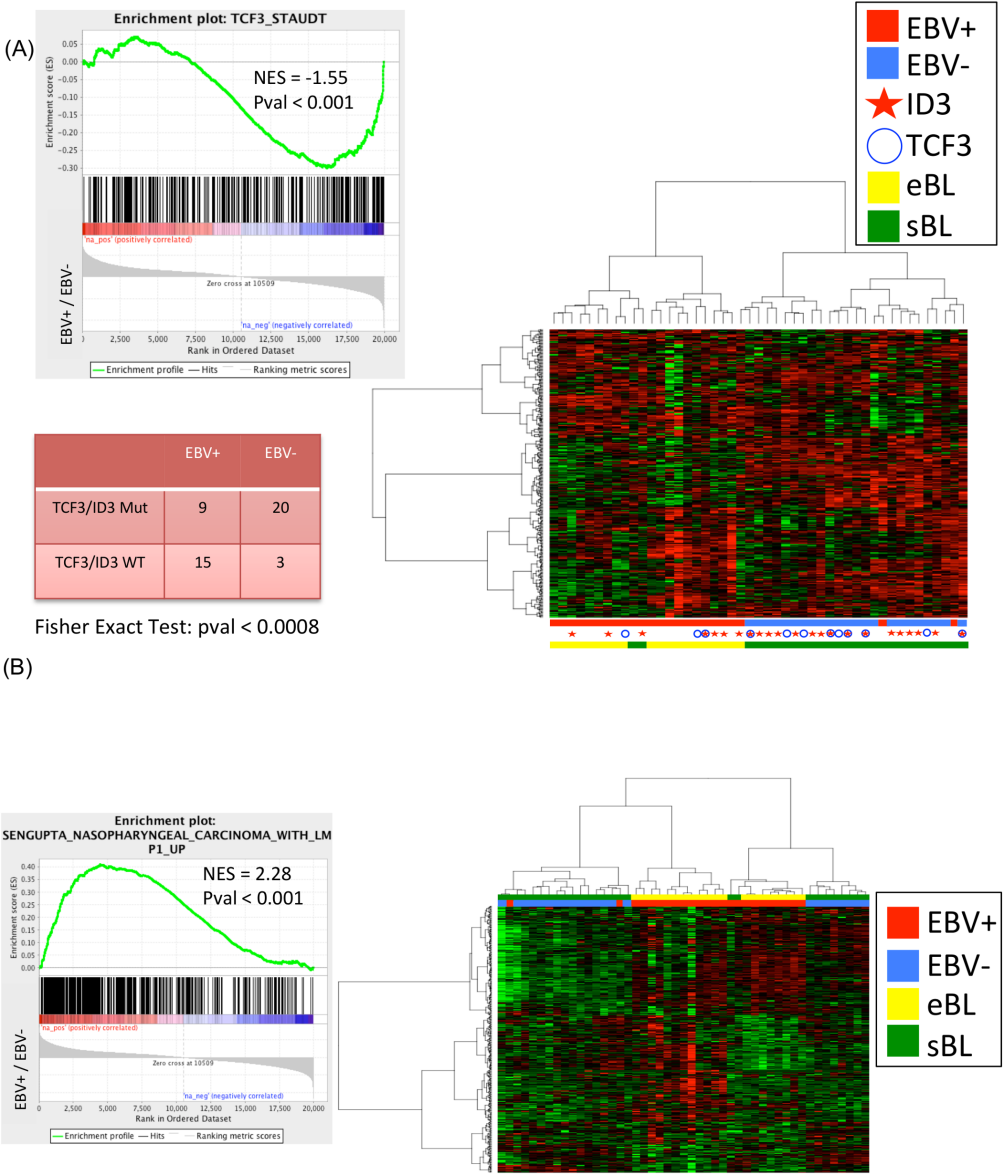
(A) the dendrogram classifies the samples into EBV-positive and EBV-negative BL independently on the specific subtype with an accuracy of 96% (45/47). (B) GSEA C2 analysis on genes differentially expressed between EBV-positive and EBV-negative cases detects a significant enrichment for the *LMP-1* gene set signature. GSEA: gene set enrichment analysis.

The distribution of somatic mutations and viral presence across both eBL and sBL samples exhibit two interesting features (Fig 3B). First, in eBL samples we observed lower mutational frequencies in the genes *MYC*, *ID3*, *TCF3*, *DDX3X*, *CCND3* and *TP53*, as compared to their reported recurrence in sBL, and higher mutational frequencies in *ARID1A*, *RHOA*, and *CCNF*[18,19]. Second, in sBL cases an almost mutual exclusivity can be seen between EBV presence and mutations in *TCF3*/*ID3* both known to be driver genes in sBL (p-value < 0.02, Fisher exact test). To explore this hypothesis, we performed a hierarchical clustering of both endemic and sporadic cases on *TCF3* target genes (previously reported in Schmitz *et al*.[19]) and we demonstrated that the first bifurcation of the dendrogram classifies the samples into EBV-positive and EBV-negative BL independently on the specific subtype with an accuracy of 96% (45/47). (Fig 4A). The results show that the TCF3 pathway is more activated in EBV-negative cases, as indicated by the significant negative enrichment of *TCF3* target genes in EBV-positive samples. Furthermore, we observe that when considering the overall panel of both endemic and sporadic BL samples, the mutually exclusivity between *TCF3*/*ID3* mutations and EBV infection yields a more significant effect (p-value < 0.0008, Fisher exact test). To further investigate the host transcriptional programming related to EBV presence, we performed GSEA C2 analysis on genes differentially expressed between EBV-positive and EBV-negative cases. Interestingly, we detected a significant enrichment for the *LMP-1* gene set signature, reported by Sengupta *et al*. [29] in nasopharyngeal carcinoma, which is consistent with the detected *LMP-1* expression in RNA-Seq data (Fig 4B). Moreover, since 13/20 RNA-Seq cases were positive for LMP2A, we investigated the role of this viral gene in the context of eBL and GSEA C2 analysis has been performed on gene differentially expressed between LMP-2A positive and LMP-2A negative samples. Interestingly, the E2F, E2F3 and cell cycle G1/S gene sets presented the highest significant enrichment score (see S11 Fig), together with the down-regulation of retinoblastoma pathway. These results can be explained as an effect of the interaction between *MYC* and LMP2A. In fact, previous studies showed that LMP-2A promotes *MYC*-induced lymphomagenesis[30], and E2F is a know target of *MYC* during cell division and proliferation[31]. Moreover, several works associate LMP-2A expression to the PI3K/Akt pathway activation[32–35] and the study from Brennan *et al*. (Oncogene, 2002 [36]) shows that the activation of PI3K pathway in lymphoblastoid cell lines can promote E2F transcription activity to affect cell cycle and cellular proliferation.

## Discussion

Over the past few years, the concept that many diseases can be etiologically linked to infection by more than one pathogen has drawn increased attention[37–40]. Whether endemic Burkitt lymphoma should also be considered a polymicrobial disease and what role genetic alterations play in the tumor are still open questions. In this paper, we analyzed the presence of pathogens other than EBV in 40 eBL primary tumors by RNA sequencing, PCR, and immunohistochemistry, and found the presence of CMV and KSHV. We detected these viruses, which are frequently reported in the African population[41], primarily in the surrounding non-neoplastic tissue. Their prevalence in areas endemic for EBV, along with their absence in the sporadic cases, suggests that CMV or KSHV could contribute to the chronic antigenic stimulation in which eBL occurs.

The presence of these additional cofactors may also induce EBV lytic cycle through B-cell reactivation and spreading EBV infection out of its natural niche of memory B-cells, characterized by a latency 0/I program[42,43]. In fact, in our samples we showed a non-canonical latency program of the virus characterized by a large number of cases expressing LMP-1/-2A/-2B in a significant proportion of cells along with lytic reactivation. Our results are in agreement with recent studies showing more complex EBV protein expression in Akata and Mutu cell lines, commonly used to study the role of EBV in Burkitt lymphoma[44]. By using an alternative approach based on RT-QPCR array platform, Tierney *et al*. report a quantitative characterization of EBV transcripts in different experimental infection models that were validated in endemic Burkitt lymphoma samples[24]. Interestingly, in this study a significant expression of *LMP-2* gene was revealed. Moreover, our results are in accordance with a previously published study in primary AIDS-related lymphomas (ARL) by Arvey and colleagues[10], although a rigorous comparison is limited by the small number of ARL BLs. All together, our findings confirm recent evidence that LMP-2A cooperates in reprogramming the function of normal B-lymphocytes and enhance *MYC* driven lymphomagenesis through the activation of PI3K-pathway[45,46]. This pathway is a crucial to *MYC* mediated transformation as shown by PI3K/MYC transgenic mouse that produces a model that represent a phenocopy of human tumors in terms of histology, gene and protein marker expression, and somatic hypermutations[47]. This scenario suggests that LMP-2A activation of PI3K is an alternative/convergent mechanism to the one driven by *TCF3*/*ID3* mutations.

The expression of genes characterizing the lytic phase of EBV found by RNA-Seq was confirmed by IHC staining for the three main genes involved in the initiation of the lytic phase, *BZLF-1/ZEBRA*, *BMRF-1/Ea-D* and *BHRF-1/Ea-R*. In early latent infection, EBV can be induced to enter the lytic cycle by a variety of causes including B-cell receptor stimulation, Toll-like receptor-9 activation, hypoxia, and growth factors[48,49]. Although lytic infection kills the host cell, it also allows horizontal spread of EBV from cell to cell and may increase the pool of latently infected B-lymphocytes from which transformed cells arise. Additionally, lytically infected B-cells secrete factors that may promote tumorigenesis, including growth and angiogenesis factors and immunosuppressive cytokines. Recent evidence has challenged the view that only the latency phase of EBV infection is significant for the development of EBV-associated malignancies, proposing that lytic EBV replication may be of pathogenic relevance.[50] Humanized mice infected with lytic active viral strains develop more lymphomas than animals infected with replication-defective strains[39], suggesting that lytic EBV infection may be of importance also in the context of an active immune response. In the present study we gave evidence for the first time that this occurs *in vivo* in the neoplastic cells of the primary tumors. Physiologically, lytic gene products are expressed in three consecutive stages: immediate-early, early, and late. Immediate-early lytic gene products initiate the process by inducing the activation of transcription of the other genes. Early genes control replication and metabolism of neoplastic cells[51]. Fatty acid synthase expression is induced by the BRLF-1 immediate–early protein, and interestingly BL tumors are characterized by altered lipid metabolism[52]. Late gene products code for viral capsid antigens and proteins involved in immune evasion. *BNLF-2A*, detected in a significant number of our cases, may protect infected B-cells from immune recognition and elimination[53]. Finally, the EBV transcriptome during the reactivation may involve the contribution of a wide array of other virus-encoded RNAs, such as BARF-0, BARF-1, BcLF-1, and RPMSI-1[54], that are not translated and may function as non-coding RNA molecules which could participate in regulating gene expression[55]. Heterogeneity in lytic/latent expression programs can be observed not only between patients but also within individual tumors, on a cell-to-cell basis. Intra-patient heterogeneity might be related to the activation of the immune response following the expression of the viral genes. Therefore, the tumor is under selective pressure and needs alternative mechanisms to survive and proliferate[56].

Our data on the mutational landscape of eBL seems to support this hypothesis. In fact, eBL samples were characterized by a lower number of point mutations in genes previously found altered in sBL, including *MYC*, *ID3*, *TCF3*, *DDX3X*, *CCND3*, and *TP53*. These results are consistent with previous studies by Schmitz *et al*. [19]in which *TCF3/ID3* mutations were more common in sBL (70% of the cases) than eBL (40%). In particular, we observed a near mutual exclusivity between *TCF3/ID3* mutations and the presence of EBV, indicating that TCF3 pathway is more significantly activated in EBV-negative cases.

The inverse correlation we observed between the presence and expression of EBV and the number of cellular mutations in the different BL cases, may represent an *in vivo* picture of the dynamic process by which a neoplastic cell, initially dependent upon EBV, switches-off viral genes and switches-on cellular mutated genes to survive and proliferate. These results are consistent with previous analysis of pediatric BL[20]. Based on our findings, one should infer that eBL may arise from pathogenic pathways that are partially distinct from those driving sBL, suggesting dual mechanisms of transformation in BL, mutationally *versus* virally driven. On the other hand, *ARID1A* and *RHOA* were more often mutated in eBL than in sBL. *ARID1A* is one of the subunits of the Switch/Sucrose Non-Fermentable (SWI/SNF) chromatin remodeling complex and is currently thought to behave like a tumor suppressor gene. Consistently, *ARID1A* mutations frequently occur as insertion/deletion, and in most of our cases involved the amino acid G1630. This gene has been reported as frequently mutated in the context of pediatric BL, with a significant association to EBV negative cases[20], suggesting that the high prevalence in eBL compared to the sBL may be due to the pediatric nature of the endemic case. However, other EBV-associated cancer types show frequent deregulation of ARID1A[57–61]. In particular, in EBV-associated gastric cancer a strong correlation between ARID1A deactivation and EBV presence has been reported[62–64]. *RHOA*, which belongs to the Ras homolog family, is a small GTP-ase protein recently found to be mutated in three tumors associated with EBV infection, namely peripheral T-cell lymphoma (where it relates to follicular helper T-cells[65–67]), diffuse gastric carcinoma[68] and paediatric sBL[28]. The distribution of *RHOA* mutations in our cohort overlaps with the already reported mutations (codons 5, 17, 42 and 69) suggesting a similar functional role.

Finally we identified recurrent mutations involving the amino acid R451C in one gene not previously detected in endemic or sporadic BLs, *CCNF*, altered in 20% of our cases. *CCNF* encodes a member of the cyclin family belonging to the F-box protein family; it acts as an inhibitor of centrosome reduplication during G2 phase and protects the cell from genome instability[69]. Therefore, it is reasonable that *CCNF* mutations may cooperate in inducing lymphomagenesis by promoting chromosome instability and a hypermutator phenotype[70,71].

Understanding the mechanisms regulating EBV lymphomagenesis will hopefully lead to the development of highly specific therapies. To avoid the tumor evasion from the already available therapies, we need to identify and target the multiplicity of pathways that are deregulated in the neoplastic cells and decrease tumor survival and proliferation.

## Materials and Methods

### Cases selection

A total of 20 BL samples preserved in RNAlater (RNA stabilization Reagent-QIAGEN, Valencia, CA) were collected from the Department of Human Pathology of the Lacor Hospital (Uganda, Africa), in endemic areas. For all of them, formalin-fixed and paraffin-embedded (FFPE) samples have been available. All diagnoses were reviewed by 2 expert hematopathologists and were formulated according to the 2008 WHO classification. The clinical and histopathologic characteristics of the 20 BL cases are summarized in S6 Table. Briefly, all cases were t(8;14)-positive, and the immunophenotype was consistent with the diagnosis of BL (CD20 positive, CD10 positive, BCL-6 positive, Ki67> 98%, BCL-2 negative). Epstein-Barr virus was detected by using *in situ* hybridization with EBER probes (INFORM EBER, Roche Diagnostics, Basel, Switzerland). EBV infection in tumor cells was observed in 100% of the samples, assessed by strong nuclear expression of small EBV-encoded RNA genes, EBER-1 and -2. These cases have been previously studied for gene expression profile analysis and showed a molecular profile consistent with molecular BL[17].

We used two distinct series of cases for validation of RNA-Seq results. The first included 26 primary tumors collected at the Moi University, Eldoret (Western Kenya). Of these, 20 were used for virus data validation and 26 for EBV latency validation. The second was comprised of 66 neoplastic samples plus 7 cases for which matched normal controls were available (1 liver, 6 lymph nodes) collected from endemic area in Africa, and was used for Sequenom validation.

### RNA extraction

Total RNA extraction was perfomed by RNeasy Plus Mini Kit(QIAGEN, Valencia, CA) according to the manufacture instructions. The amount and quality of RNA were evaluated by measuring the optical density (OD) at 260 nm, the 260/230 and the 260/280 ratios using a Nanodrop spectrophotometer (ND-100, Nanodrop, Thermo Scientific, Celbio, Italy).

### RNA sequencing

Paired-end libraries (2x75 base pair) were prepared according to the TruSeq RNA sample preparation v2 protocol (Illumina, San Diego, USA). Briefly, 2 μg of Poly(A)^+^ RNA was purified from total RNA using poly-T oligo attached magnetic beads and then used for fragmentation into 130–290 bp fragments. First, single stranded cDNA was synthesized using reverse transcriptase (SuperScript II, Invitrogen, Life Technologies,USA) and random hexamer priming, followed by generation of double-stranded cDNA. AmpureXP beads (Beckman Coulter, Brea CA) were used to purify the ds cDNA and end repair step was performed to convert the overhangs, resulting from fragmentation, into blunt ends by 3’ to 5’ exonuclease activity. A single “A” nucleotide was added to the 3’ ends of the blunt fragments to prevent them from ligating to one another during the adapter ligation reaction. This approach was adopted to ensure a low rate of chimera (concatenated template) formation. Subsequently, sequencing adapters were added to the ends of the ds cDNA fragment and a PCR reaction was used to selectively enrich those ds cDNA fragments that had adapter molecules on both ends, amplifying the amount of ds cDNA in the final libraries. Lastly, PCR library products were purified by AmpureXP beads and quality control analysis was assessed using a DNA-1000 (Agilent, USA). The quantification was performed by Quant-it PicoGreendsDNA Assay Kit according to manufacturer’s protocol (Invitrogen, Life Technologies,USA). The resulting libraries were sequenced on an Illumina HiScan SQ (Illumina, San Diego, USA) following the manufacturer's instructions.

### Point mutation identification using RNASeq

Sequence variants were obtained using the SAVI (Statistical Algorithm for Variant Identification)[72,73] algorithm independently for each sample. Candidate somatic mutations were obtained by eliminating common germline variants (dbSNP 132 and variants from 10 reactive lymph nodes). Genes recurrently mutated in more than 4 samples and expressing the corresponding transcript with RPKM>3 were selected. Mutations occurring in the exact same position in more than 4 samples have been discarded. Conversely, genes previously reported in BL[19,74,75] were selected, even at low recurrence, to allow the comparison between endemic and sporadic subtypes (S3 Table). Sanger sequencing was used for technical validation.

### Pandora: A pipeline for pathogen discovery using RNASeq

Characterization of the tumor microbiome is accomplished with Pandora, a new RNA-Seq pipeline for pathogen identification and discovery (S12 Fig). The algorithm takes raw RNA-Seq data as input and outputs annotated microbial spectra present in the tumor sample. Pandora implements a subtractive algorithm consisting of discrete modules. First, the *Host Removal* phase sequentially aligns the input reads to the host reference using bowtie2[76], blastN[77] and Megablast,[78] and filters out the data originating from the host. Second, the unaligned (non-host) reads are passed as input to the *Microbe Identification* phase where the reads are aligned to curated sets of NCBI microbial sequences representing viruses/viroids, bacteria, fungi, and select taxa of eukaryotic parasites. Third, the NCBI records matching each non-host read are input to the *Reporting* phase where microbial load, gene expression, and relevant clinical parameters are computed as the final output. The microbial load is computed as the number of reads mapping to the organism or virus normalized by the genome length. Gene expression quantification is computed as transcript per million (TPM)[79], which provides a more accurate relative quantification of mRNA abundance compared to other normalization methods such as RPKM.

### EBV genome analysis and expression

RNA-Seq reads were aligned to the GRCh37/hg19 reference genome using Bowtie2[76], Blastn[77] and Megablast[78]. Reads not aligning to homo sapiens (non-host reads) were mapped to human herpesvirus 4, type I (NCBI accession number NC_007605.1) using TopHat, a splicing aware alignment program [80] (S9 Table and S10 Table). EBV viral gene expression was normalized as transcripts per million (TPM)[79]. For each viral product the TPM expression was normalized by the expression of A73 genes, which is consistently expressed in all the HHV4 positive BL samples. Hierarchical clustering was computed with Pearson distance and Ward’s linkage method.

### Expression analysis

Gene expression analysis was performed on both endemic and sporadic[19] RNA-Seq samples of Burkitt Lymphoma. All the reads were aligned to human reference genome (GRCh37/hg19) by means of TopHat version 1.3.3. Transcript abundance quantification was computed as FPKM using Cufflinks, Cuffquant and Cuffnorm version 2.2.1[81]. Hierarchical clustering was performed with Pearson distance and Ward’s linkage method. Gene set enrichment analysis was obtained by running GSEA software on pre-ranked list of log2 ratio of the FPKM mean fold change between two conditions[82].

### DNA extraction

The DNA was extracted from formalin-fixed paraffin embedded (FFPE) of the original neoplastic samples using NucleoSpin Tissue (Machery-Nagel, Italy) following manufacture’s instructions. The amount and quality of DNA were evaluated by measuring the optic density (OD) at 260 nm, the 260/230 and the 260/280 ratios using a Nanodrop spectrophotometer (ND-100, Nanodrop, Thermo Scientific, Celbio, Italy).

### PCR amplification

To detect the presence of HTLV-1, CMV and KSHV, a nested PCR assay was performed on DNA of original tumor samples as previously reported[83]^,^[84] (S7 Table and S8 Table). DNA from HTLV-1-positive cells, CMV-positive cells, and KSHV-positive cells were used as positive controls, whereas DNA from HeLa293 cells was used as negative control. Several precautions have been taken to prevent false-positive PCR results: (a) rooms for pre- and post-PCR procedures were physically separated; (b) reagents were prepared in large batches and stored in small aliquots; (c) equipment such as the microcentrifuge, water baths, pipettes, tube racks, and other small equipment was designated for PCR work only; (d) gloves were changed frequently; and (e) aerosol-barrier pipette tips, PCR tubes, and autoclaved, diethylpyrocarbonate-treated water were sterilized by UV irradiation prior to PCR. Finally, 15 μl aliquots of the PCR mixture were electrophoresed on a 2% agarose gel and directly visualized by ethidium bromide staining under ultraviolet light[85].

### Validation in an extended panel

The MassARRAY Assay Design Suite software was used to design 8 different multiplex reactions for investigating 115 SNPs. Genotyping was performed using iPLEX Gold technology 57 MassARRAY high-throughput DNA analysis with matrix-assisted laser desorption/ionization time-of-flight mass spectrometry (Sequenom), according to the manufacturer’s protocol. 66 neoplastic cases plus 7 samples with matched normal controls (1 liver, 6 lymph nodes) were analysed.

### Real-time quantitative reverse transcription PCR (RT-qPCR)

The expression of EBV-encoded genes (*EBNA-1*, *EBNA-2*, *EBNA-3c*, *BALF-2*, *BALF-4*, *BCRF-1*, *BHRF-1*, *BILF-1*, *BNLF-2a*, *BMRF-1*, *BZLF-1*, *LMP-1*, *LMP-2A LF-2*), which characterize the different latency programs, has been investigated on an additional series of 26 samples by RT-qPCR using the QuantiTect SYBR Green PCR Kit (Qiagen, CA) as previously reported (S7 Table). All samples were run in triplicate. The stably expressed housekeeping gene hypoxanthine-guanine phosphoribosyltransferase (*HPRT*) was used as an endogenous control and reference gene for relative quantification of each target gene. The relative expression is expressed as 2^ΔCt^, where ΔCt is defined as the difference in mean cycle thresholds of the gene of interest and HPRT[86]. The samples were defined as “not expressed” if the ΔCt value exceeded 50 cycles[87].

### Immunohistochemistry

To further validate the presence of HTLV-1 and HHV-8, immunohistochemistry for viral products (HTLV-1-TAX 1: 70, Abcam, Cambridge, United Kingdom; HHV8-LANA 1: 50, Leica Biosystems, Newcastle Lid, United Kingdom; HHV8 clone AT4C11 1:50, Abnova, Taipey City, Taiwan) was performed on formalin-fixed paraffed-embedded (FFPE) sections of the original samples and in an additional series of 20 cases. CMV was detected using *in situ* hybridization (ISH) with Bond ISH Probe.

The protein expression of EBNA-1 (1:150, AbCam, Italy), EBNA-2 (1:100, AbCam, Italy), LMP-1 (1:100, Novus Biologicals, Italy), LMP-2A (1:100, AbCam, Italy), BZLF-1/ZEBRA (1:100, Novus Biologicals, Italy), BMRF-1/Ea-D (1:150, AbCam, Italy), BHRF-1/Ea-R (1:150, Novus Biologicals, Italy), was assessed by immunohistochemistry on FFPE sections of the original samples and on an additional series of 26 primary tumors. Sections of the samples incubated with the secondary antibody alone and sections of reactive lymphoid tissue were used as negative controls. Sections of lymph nodes with infectious mononucleosis were used as positive control. Immunoreactivity was performed on Bond Max automated immunostainer (Leica Microsystem, Bannockburn, IL, USA), with controls in parallel. No epitope retrieval was used. Ultravision Detection System using anti-Polyvalent HRP (LabVision, Fremont, CA, USA) and diaminobenzidine (DAB, Dako, Milan-Italy) as chromogen was used. Two independent investigators assessed immunoreactivity. Case were considered positive when more than 20% of the cells were stained for latent gene products and when more than 5% of the cells were stained for lytic gene products.

### Ethics statement

Ethics approval for this study was obtained from the Institutional Review Board at the University of Siena (Italy), from the Ethics and Research Committee at the Lacor Hospital (Uganda) and from the Ethics and Research Committee at Moi University, Eldoret (Kenya). Written permission and informed consent have been obtained before sample collection in accordance with the Declaration of Helsinki.

## Supporting Information

S1 Fig Number of viral reads per million of human reads across the 20 eBL of the discovery cohort. The red line indicates the minimal number of viral reads to detect any of the viruses in the corresponding sample.
(TIFF)

S2 Fig CMV detection on discovery cohort samples by IHC. (TIFF)

S3 Fig KSHV detection on discovery cohort samples by IHC. (TIFF)

S4 Fig CMV detection on Kenyan cohort samples by IHC. (TIFF)

S5 Fig KSHV detection on Kenyan cohort samples by IHC. (TIFF)

S6 Fig The specific analysis of EBV isoforms showed the presence of H2-HF splicing event. (TIFF)

S7 Fig Validation of latency and lytic genes expression by RT-qPCR for *LMP-2A (A)*, *BZLF-1 (B)*, *BMRF-1 (C)*, *BHRF-1* (D) in additional series of 26 cases is shown. (TIFF)

S8 Fig A-B Results of Sequenom analysis on an extended panel of 66 neoplastic samples *plus* 7 cases with matched normal controls are demonstrated. (TIFF)

S9 Fig A-C Examples of mutations in *RHOA* (A-B) and in *CCNF* (C) detected by Sequenom technology and validated by Sanger sequencing are shown. (TIFF)

S10 Fig A-F Examples of mutations in *ARID1A* (A), DDX3X (B), *CCNF* (C), *RHOA* (D), *ID3* (E), *TCF3* (F) detected by RNA-Seq technique and validated by Sanger sequencing are shown. (TIFF)

S11 Fig A-B The E2F, E2F3 and cell cycle G1/S gene sets presented the highest significant enrichment score together with the down-regulation of retinoblastoma pathway. (TIFF)

S12 Fig Characterization of the tumor microbiome by Pandora. (TIFF)

S1 Table (A) PCR Nested and IHC validation results on original samples. (B) IHC validation results on extended cohort from Kenya. (TIFF)

S2 Table Validation of latency type by RT-qPCR in comparison with RNA-sequencing results. (TIFF)

S3 Table Immunohistochemical results in single case. (TIFF)

S4 Table List of selected mutations detected in RNA-Seq. (XLSX)

S5 Table List of variants detected through Sequenom technology. (XLS)

S6 Table Clinical and histopathologic features of discovery cohort BL patients. (TIFF)

S7 Table Primers sequences for detection and typing of human lymphotropic herpesviruses. (TIFF)

S8 Table Primers sequences for detection of EBV-encoded genes. (PDF)

S9 Table Complete list of mutations detected in RNA-Seq. (XLSX)

S10 Table Table of read counts of all the human genes and transcripts detected in RNA-Seq. (XLSX)
