# Supplementary figures and images for: Distinct Viral and Mutational Spectrum of Endemic Burkitt Lymphoma
Source: PLoS Pathog. 2015 Oct 15;11(10):e1005158. doi: 10.1371/journal.ppat.1005158 (PMC4607508; doi:10.1371/journal.ppat.1005158)

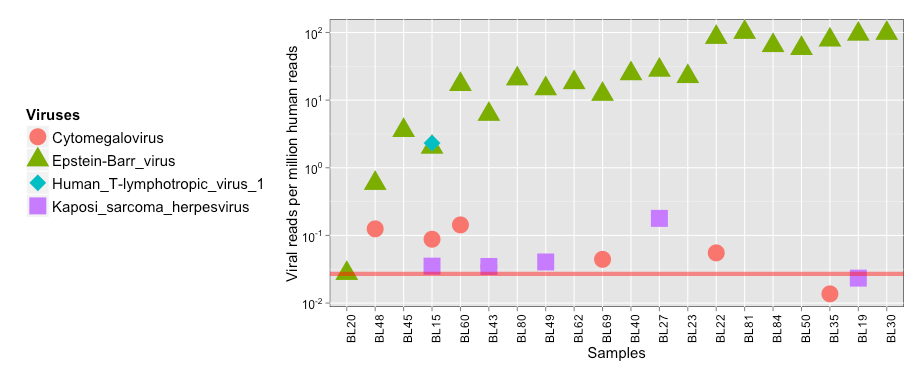

Supplement: S1 Fig — The red line indicates the minimal number of viral reads to detect any of the viruses in the corresponding sample. (TIFF) [file ppat.1005158.s001.tiff]

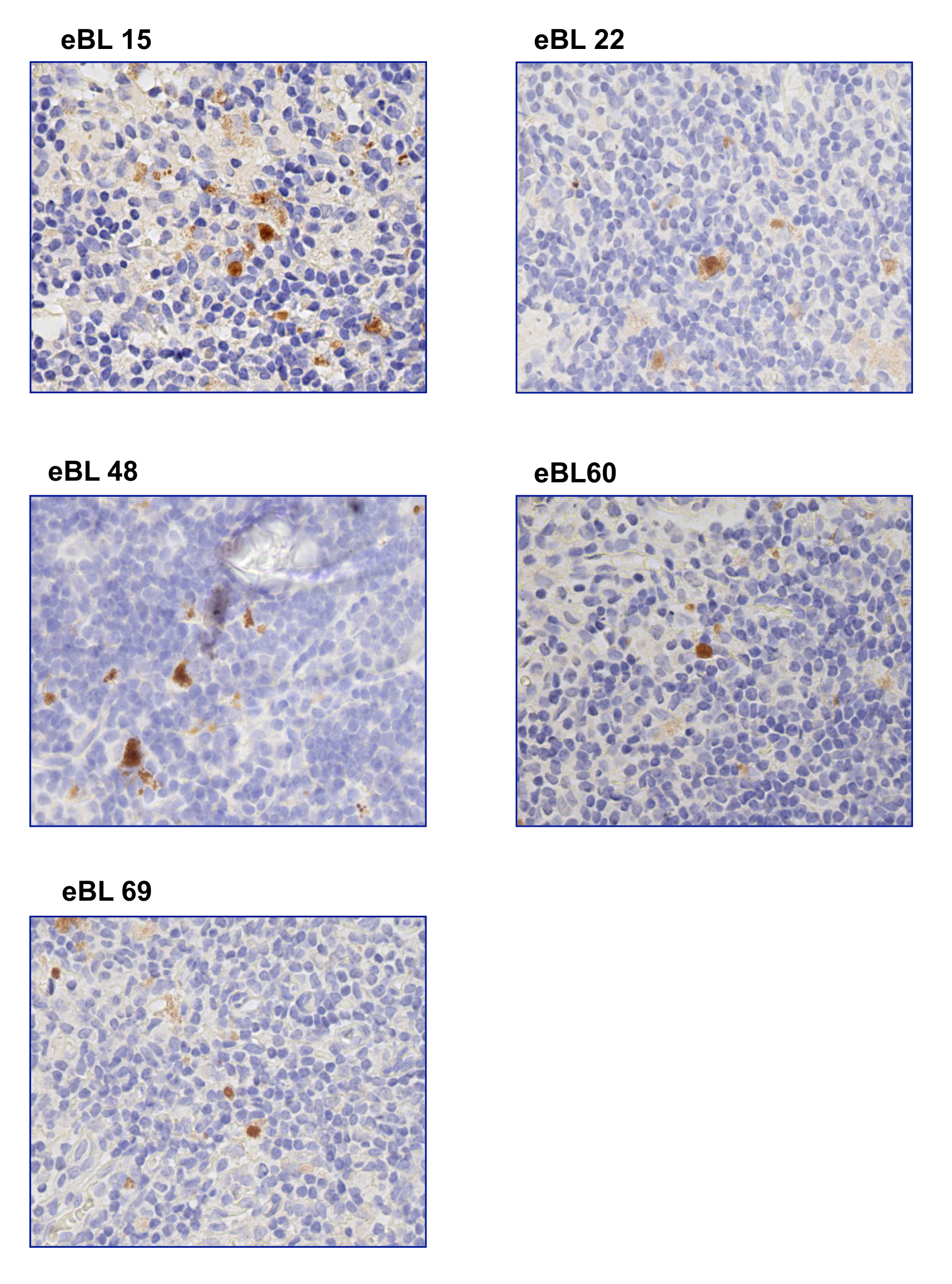

Supplement: S2 Fig — (TIFF) [file ppat.1005158.s002.tiff]

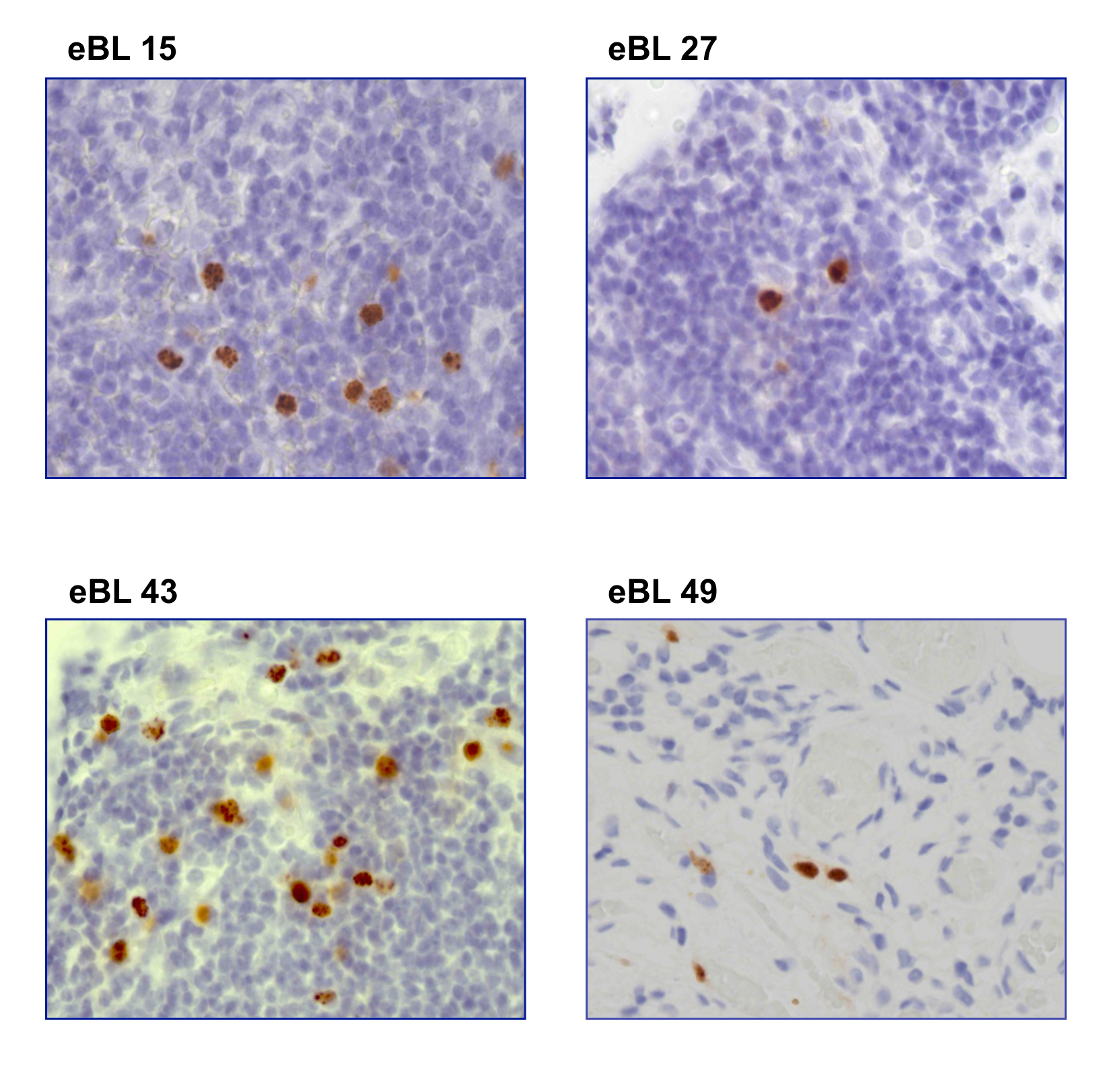

Supplement: S3 Fig — (TIFF) [file ppat.1005158.s003.tiff]

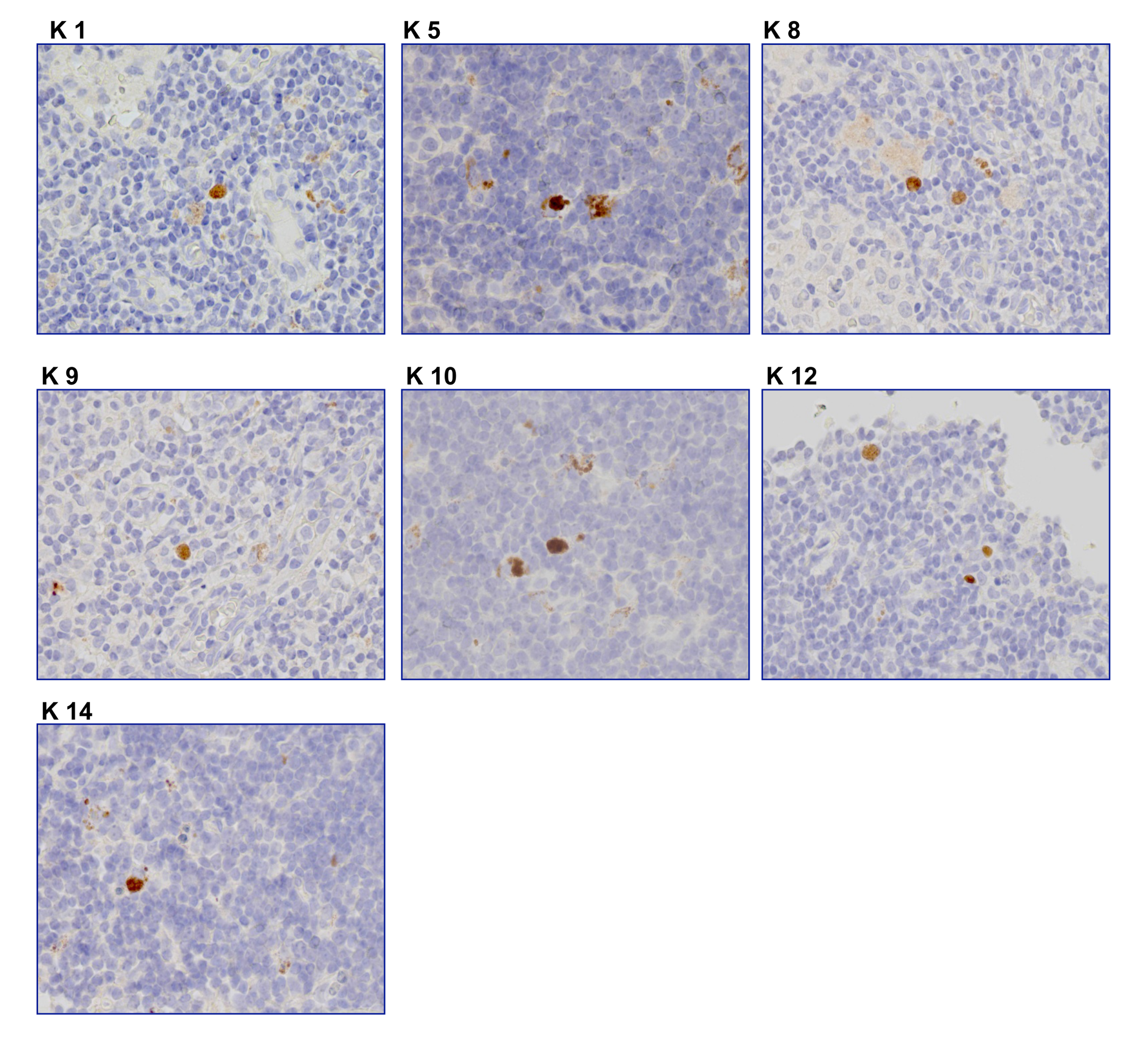

Supplement: S4 Fig — (TIFF) [file ppat.1005158.s004.tiff]

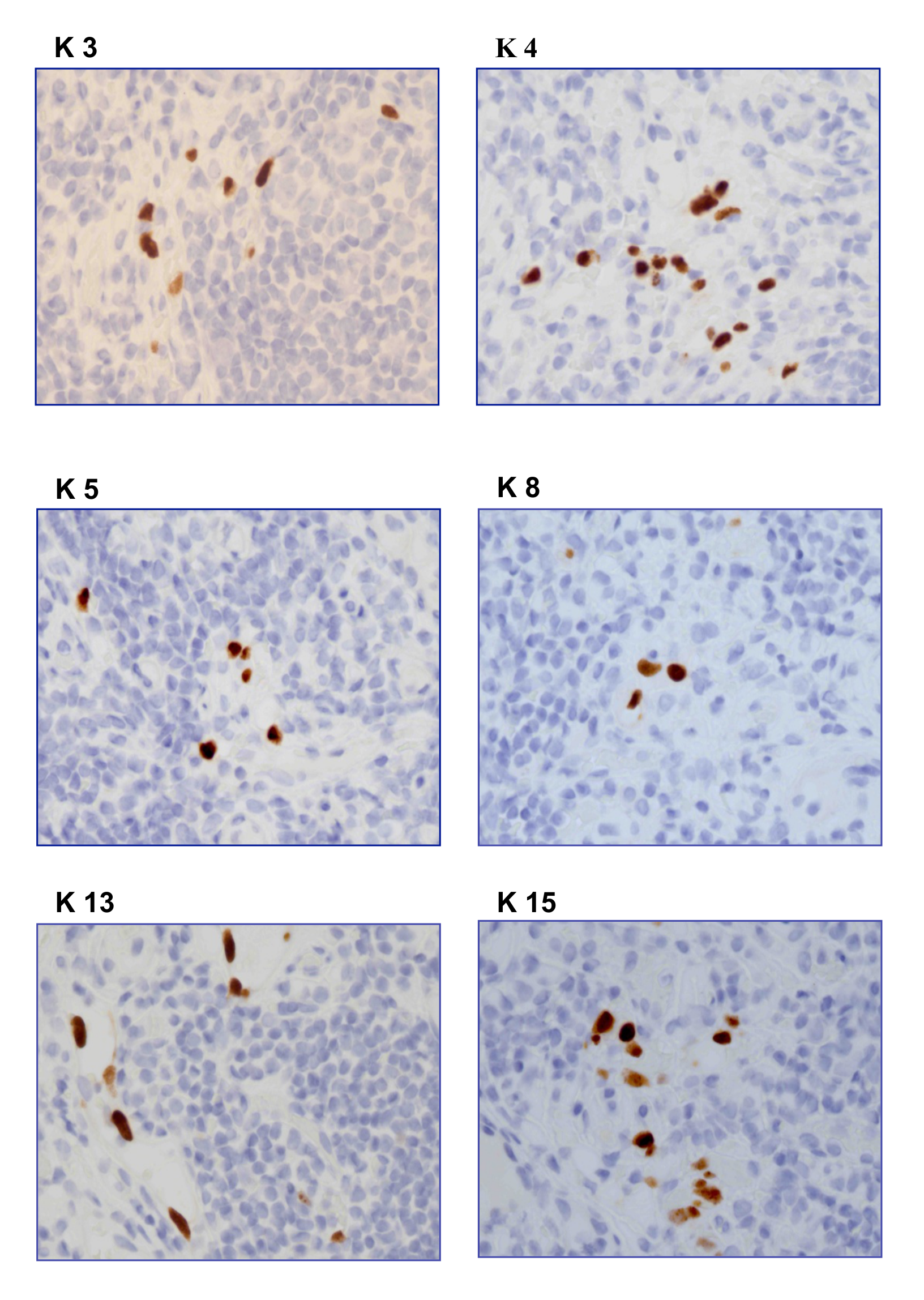

Supplement: S5 Fig — (TIFF) [file ppat.1005158.s005.tiff]

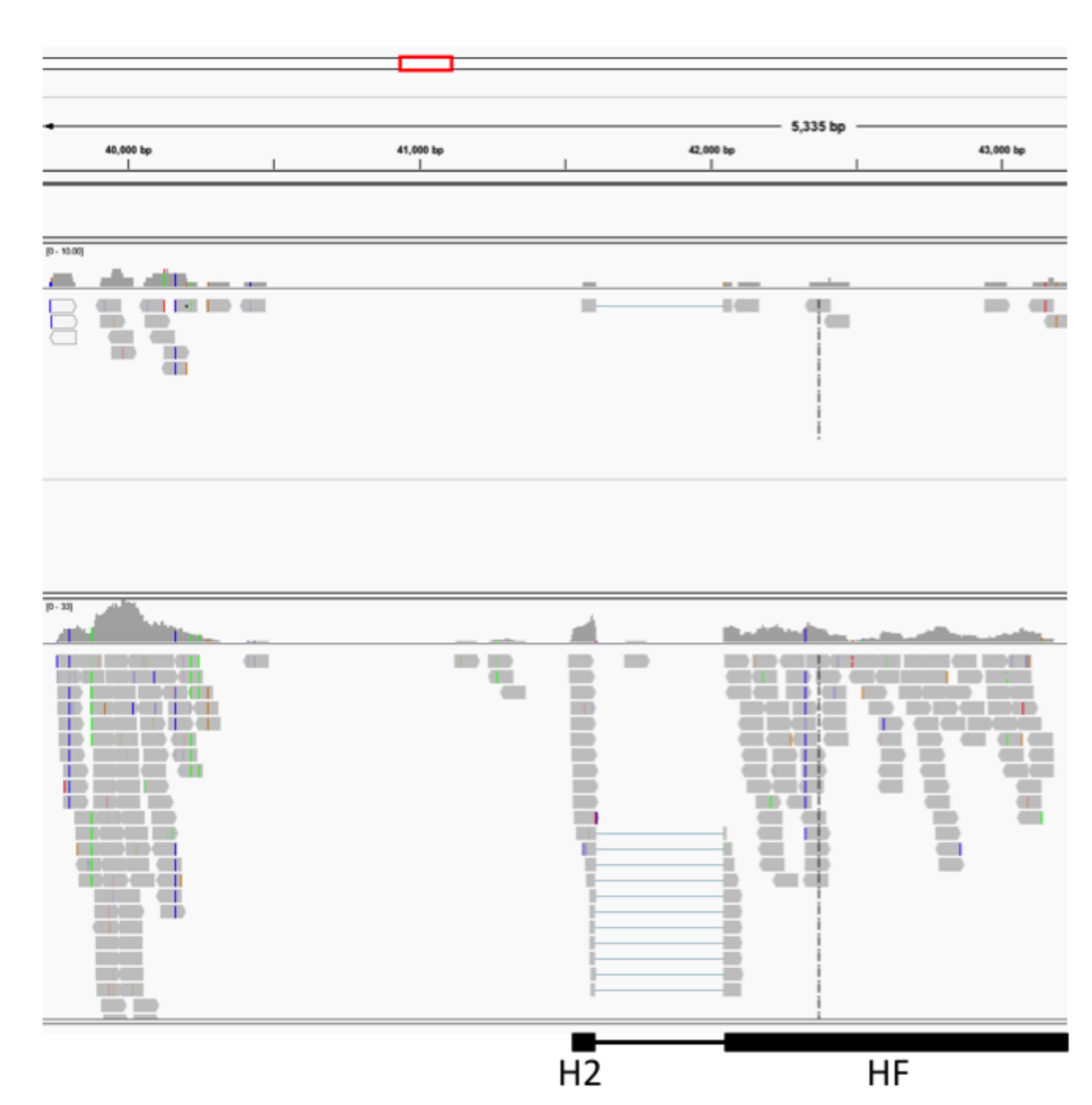

Supplement: S6 Fig — (TIFF) [file ppat.1005158.s006.tiff]

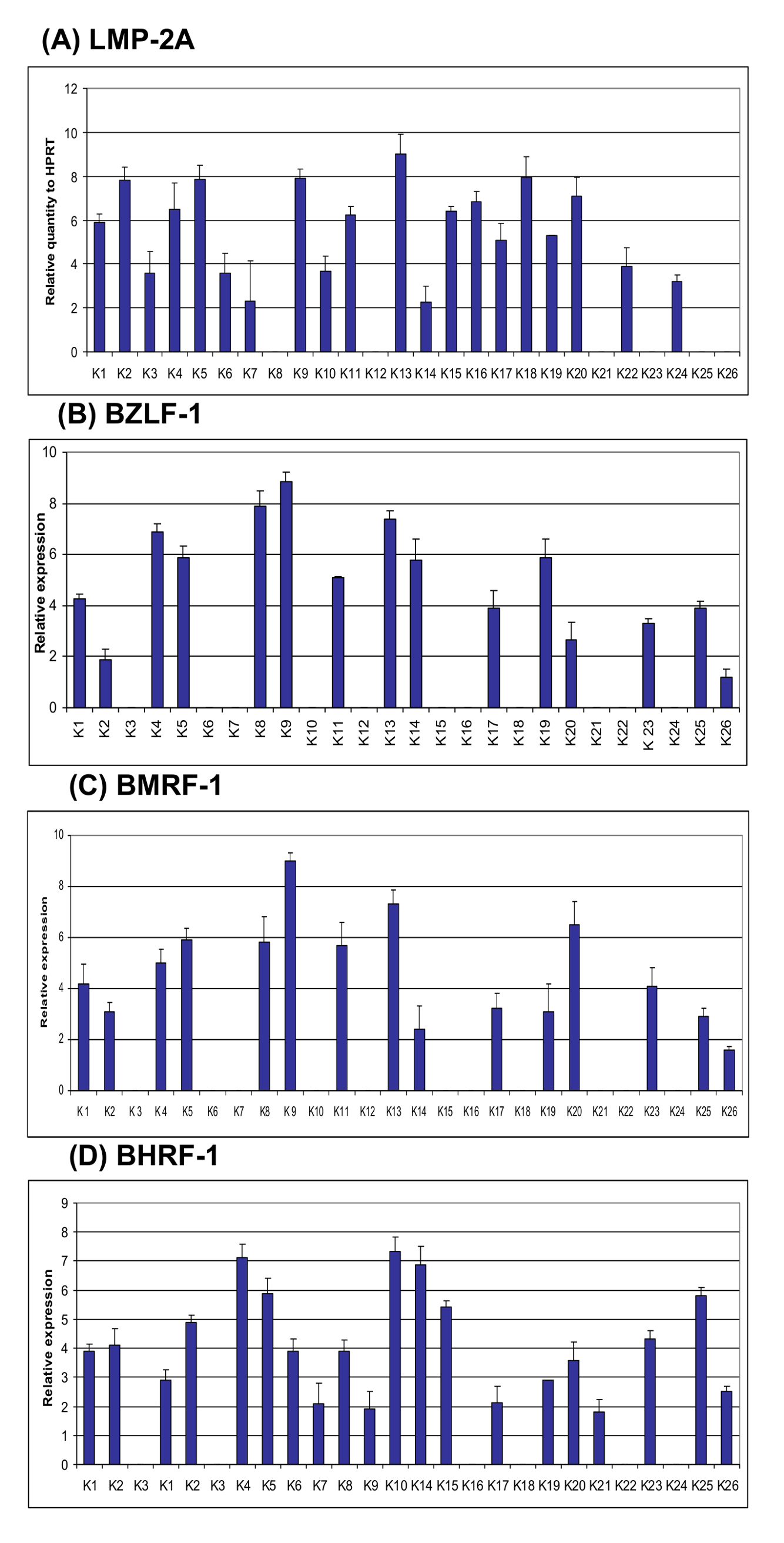

Supplement: S7 Fig — (TIFF) [file ppat.1005158.s007.tiff]

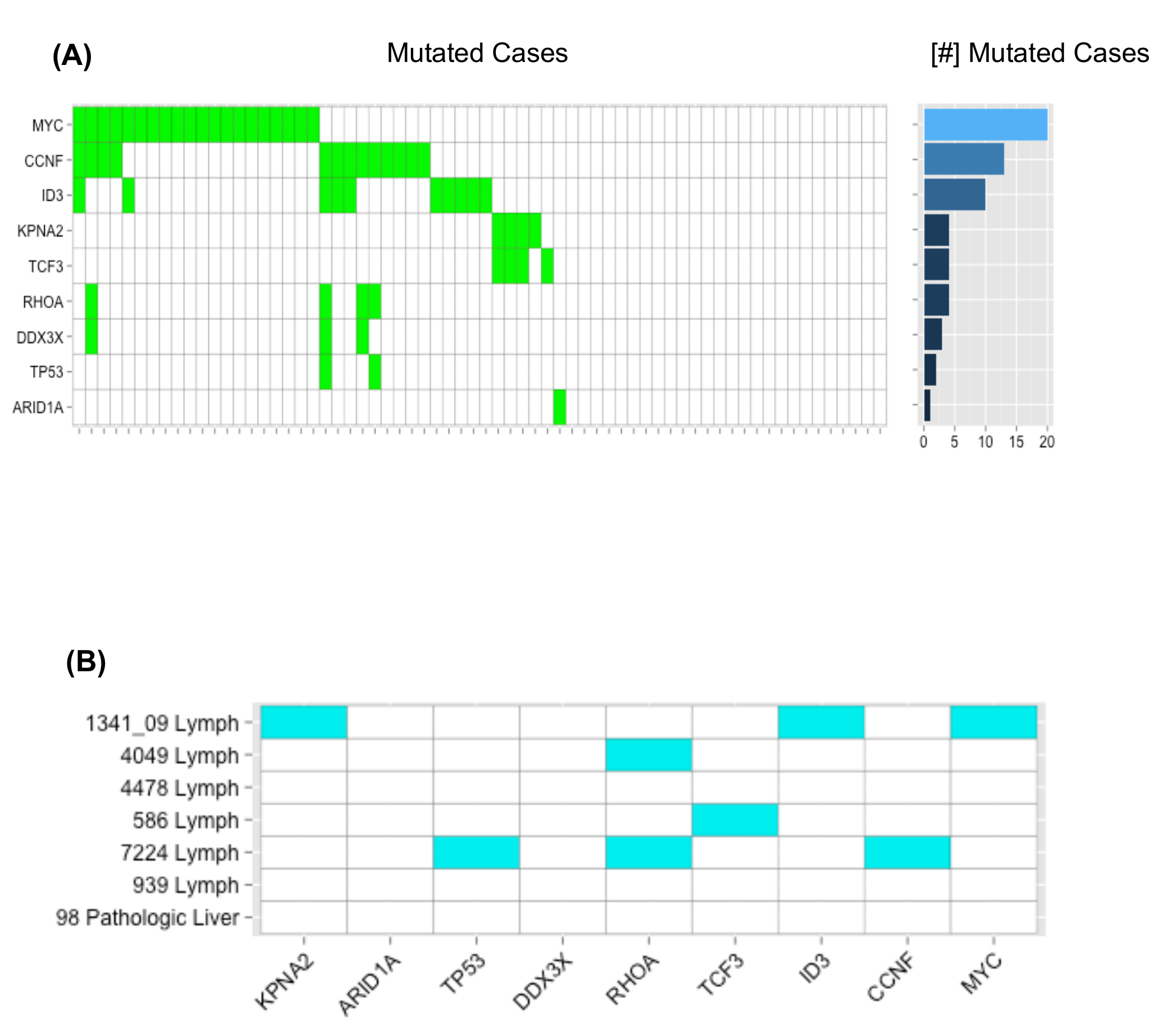

Supplement: S8 Fig — (TIFF) [file ppat.1005158.s008.tiff]

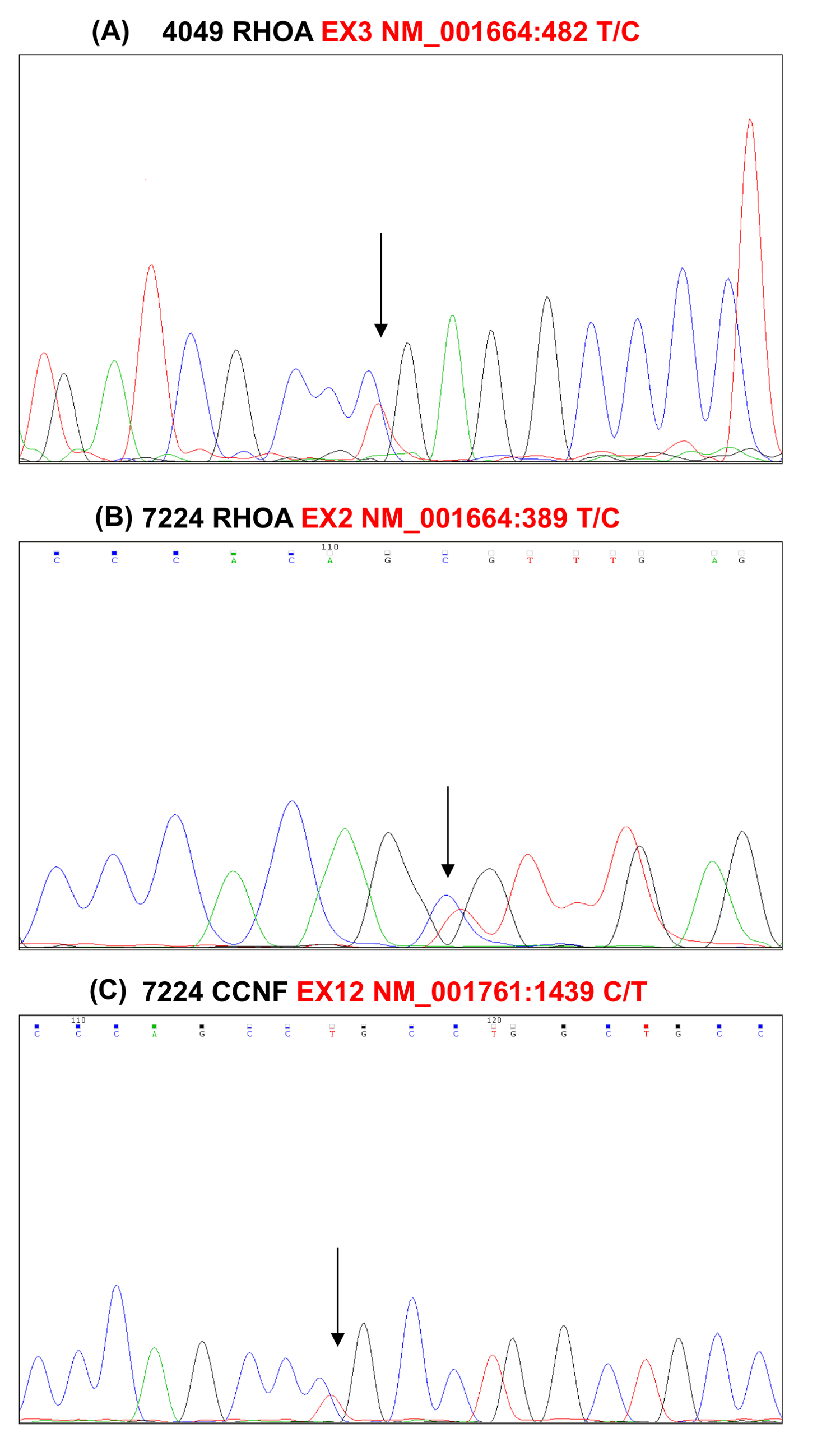

Supplement: S9 Fig — (TIFF) [file ppat.1005158.s009.tiff]

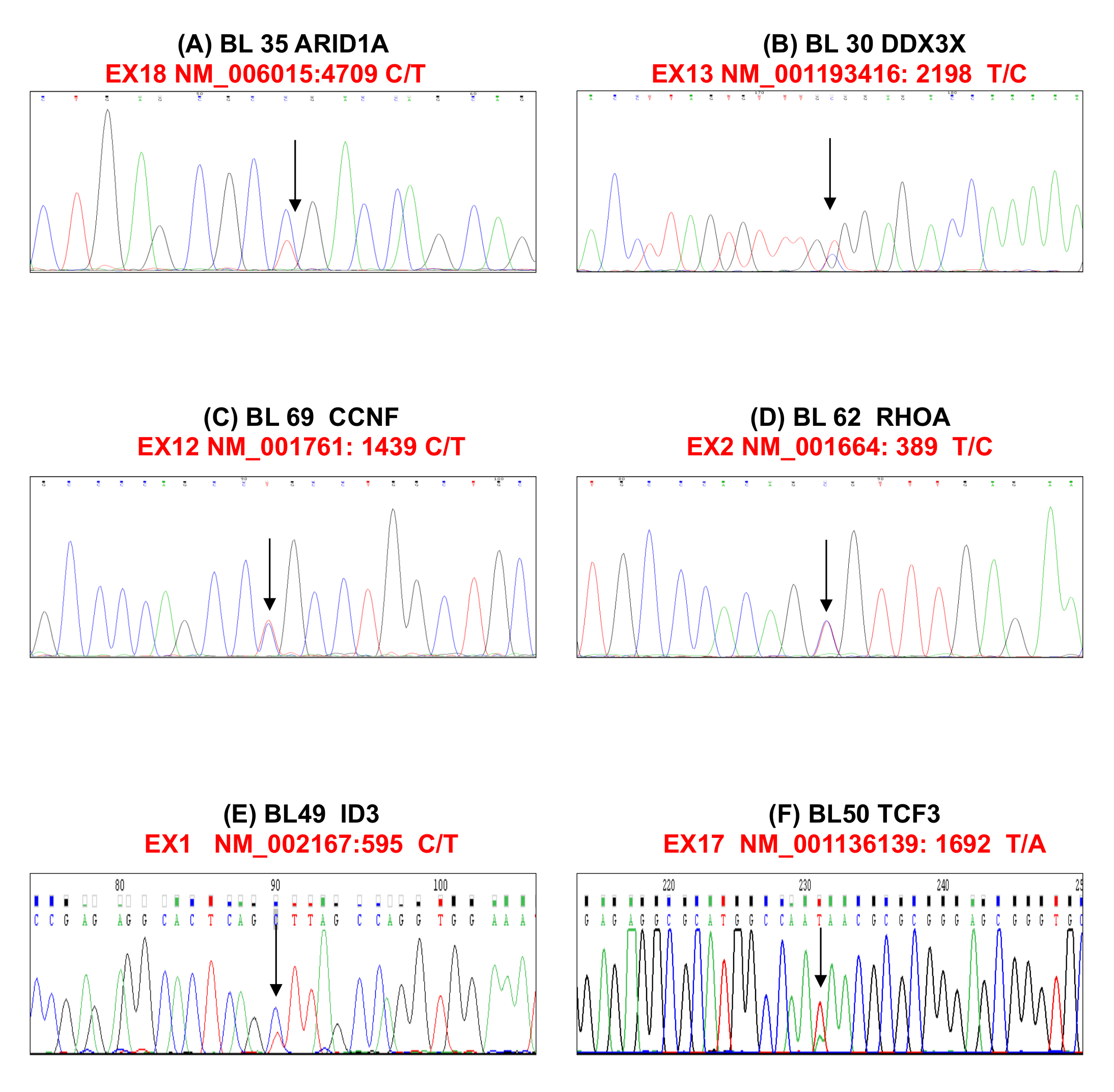

Supplement: S10 Fig — (TIFF) [file ppat.1005158.s010.tiff]

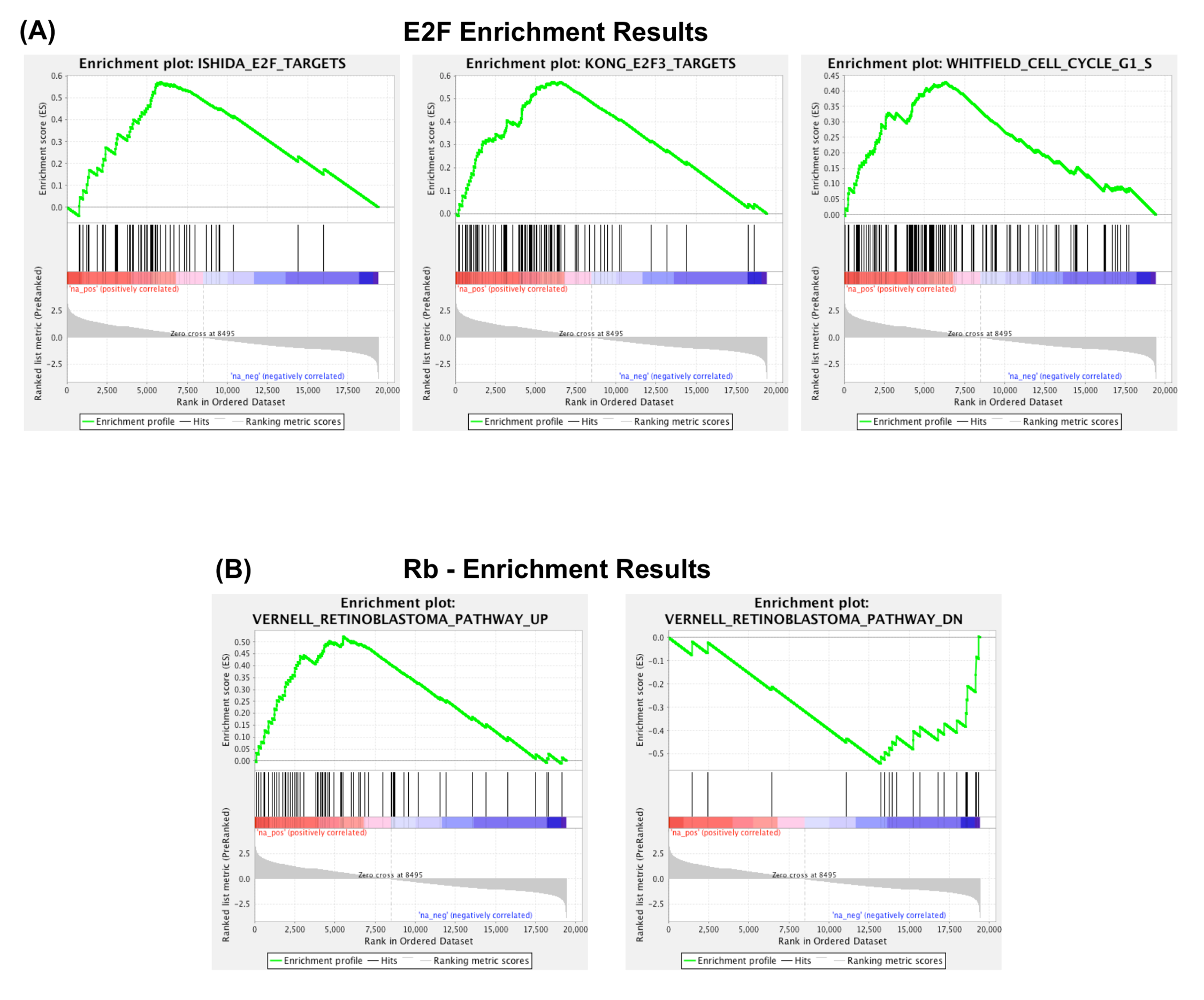

Supplement: S11 Fig — (TIFF) [file ppat.1005158.s011.tiff]

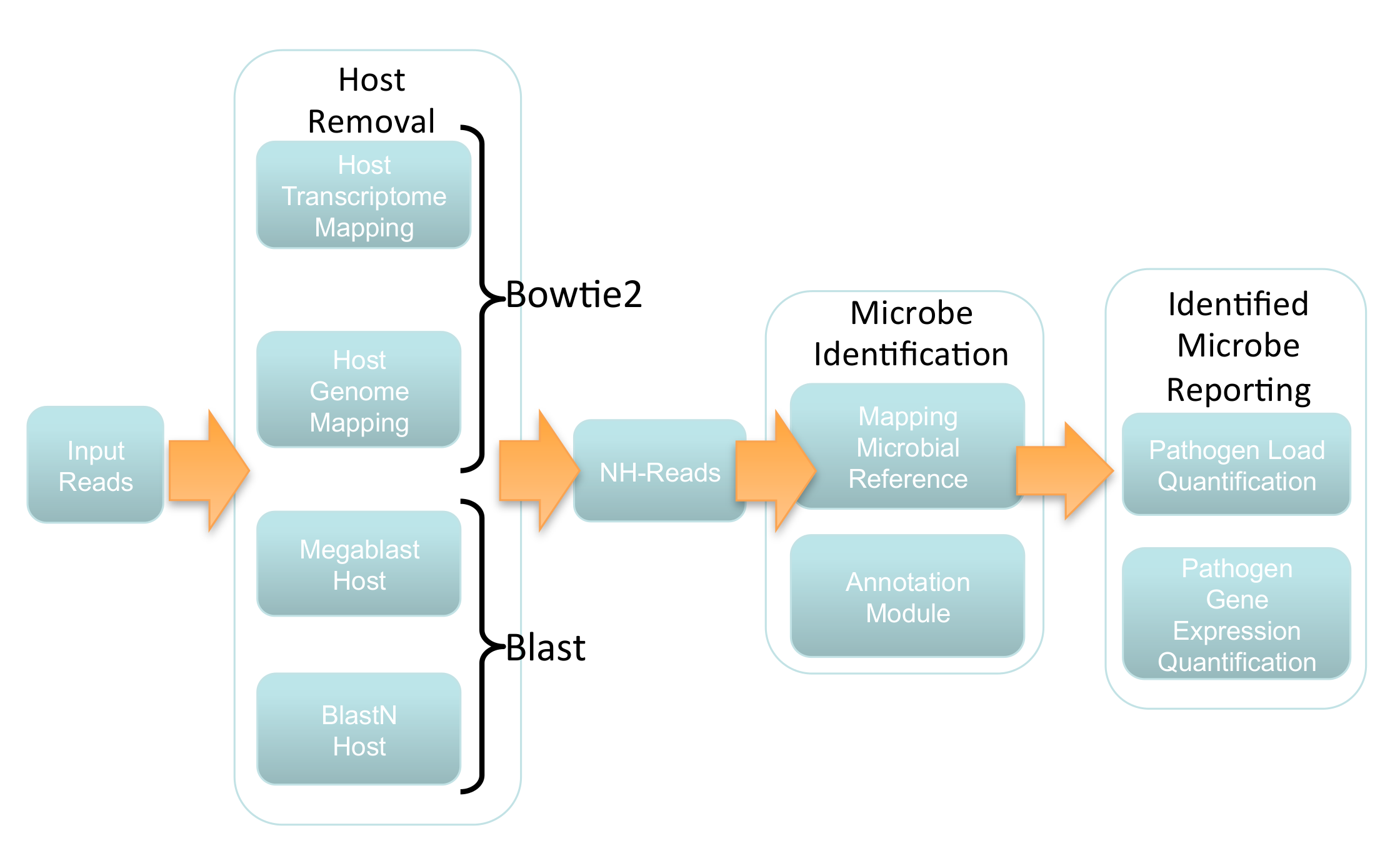

Supplement: S12 Fig — (TIFF) [file ppat.1005158.s012.tiff]

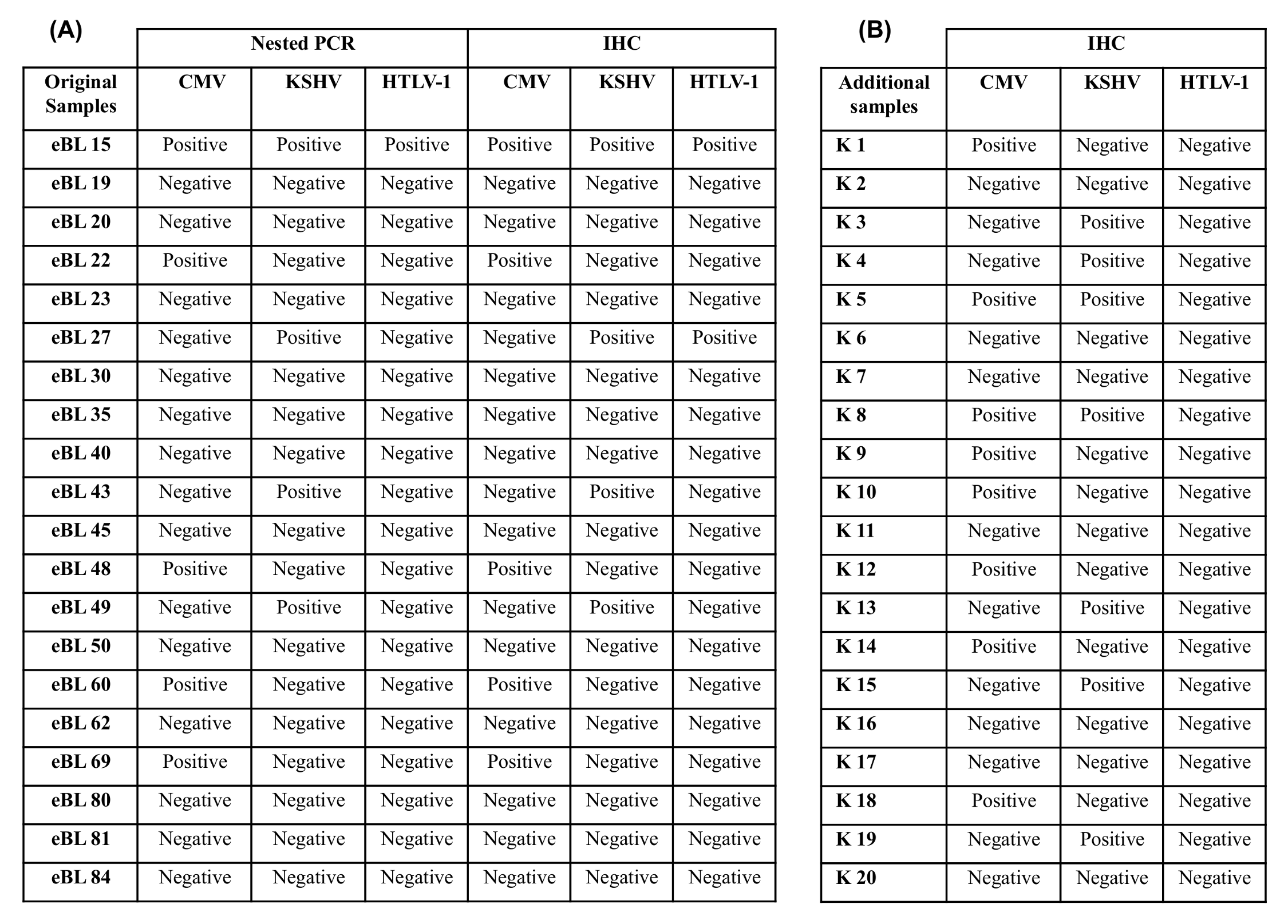

Supplement: S1 Table — (TIFF) [file ppat.1005158.s013.tiff]

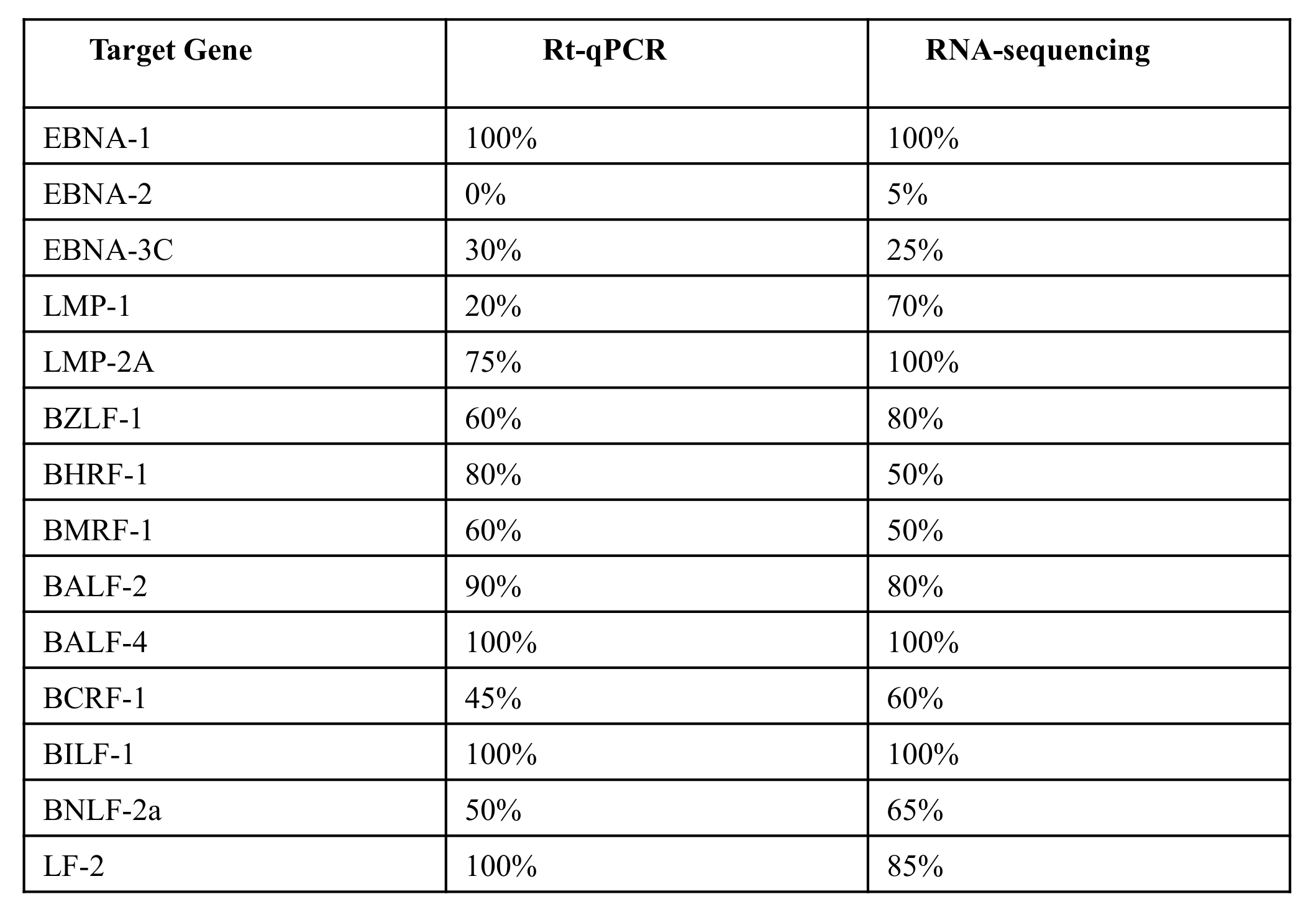

Supplement: S2 Table — (TIFF) [file ppat.1005158.s014.tiff]

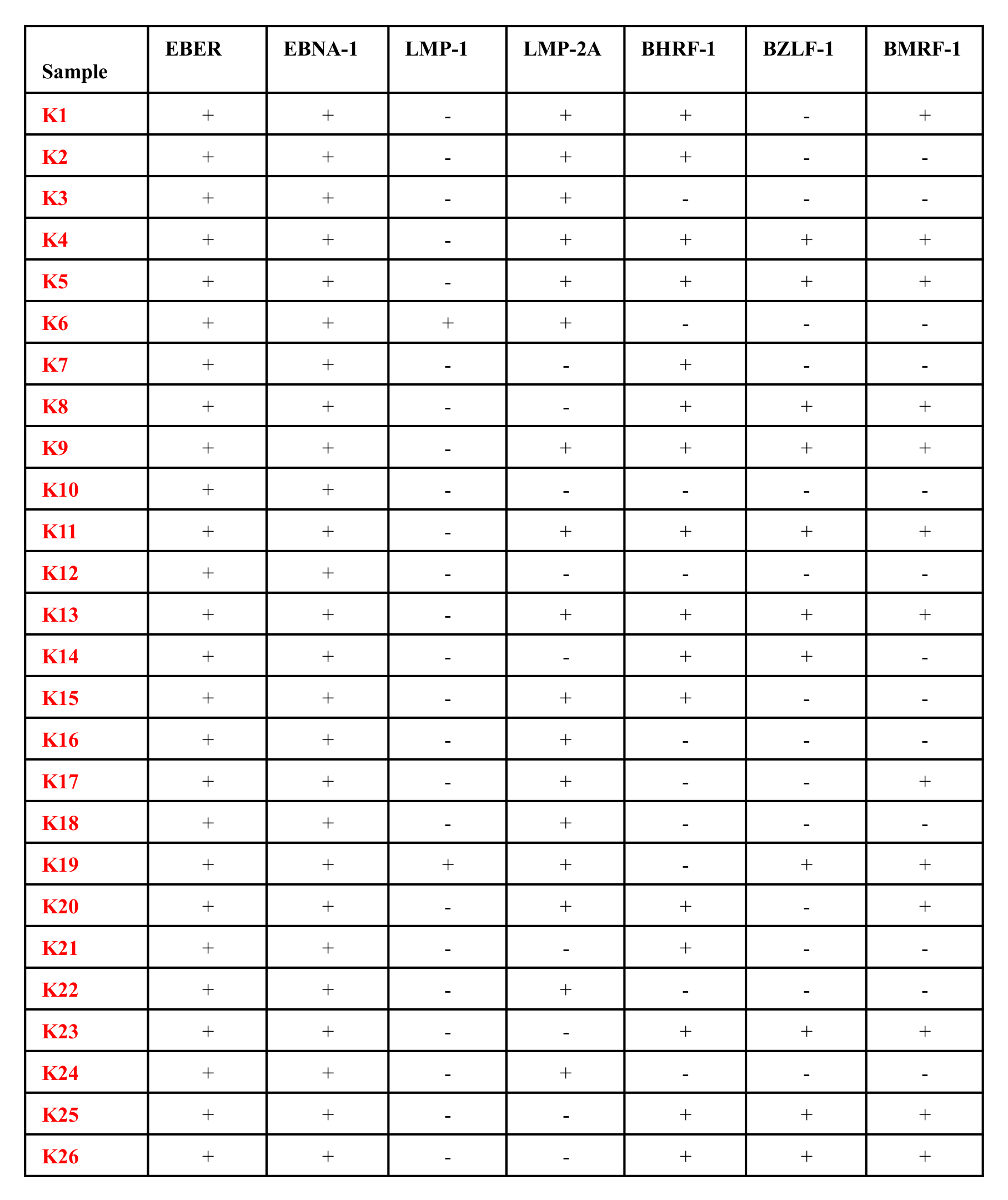

Supplement: S3 Table — (TIFF) [file ppat.1005158.s015.tiff]

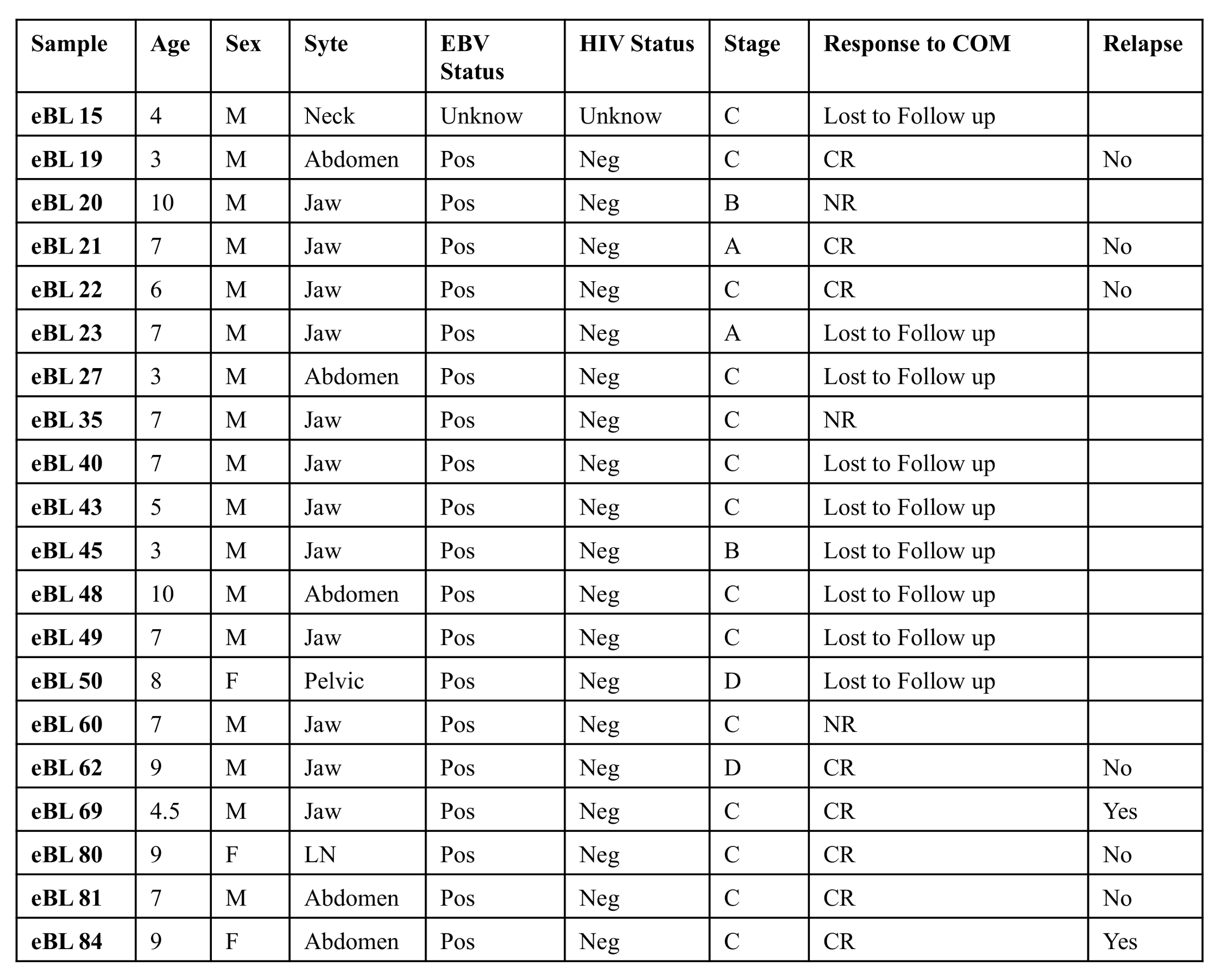

Supplement: S6 Table — (TIFF) [file ppat.1005158.s018.tiff]

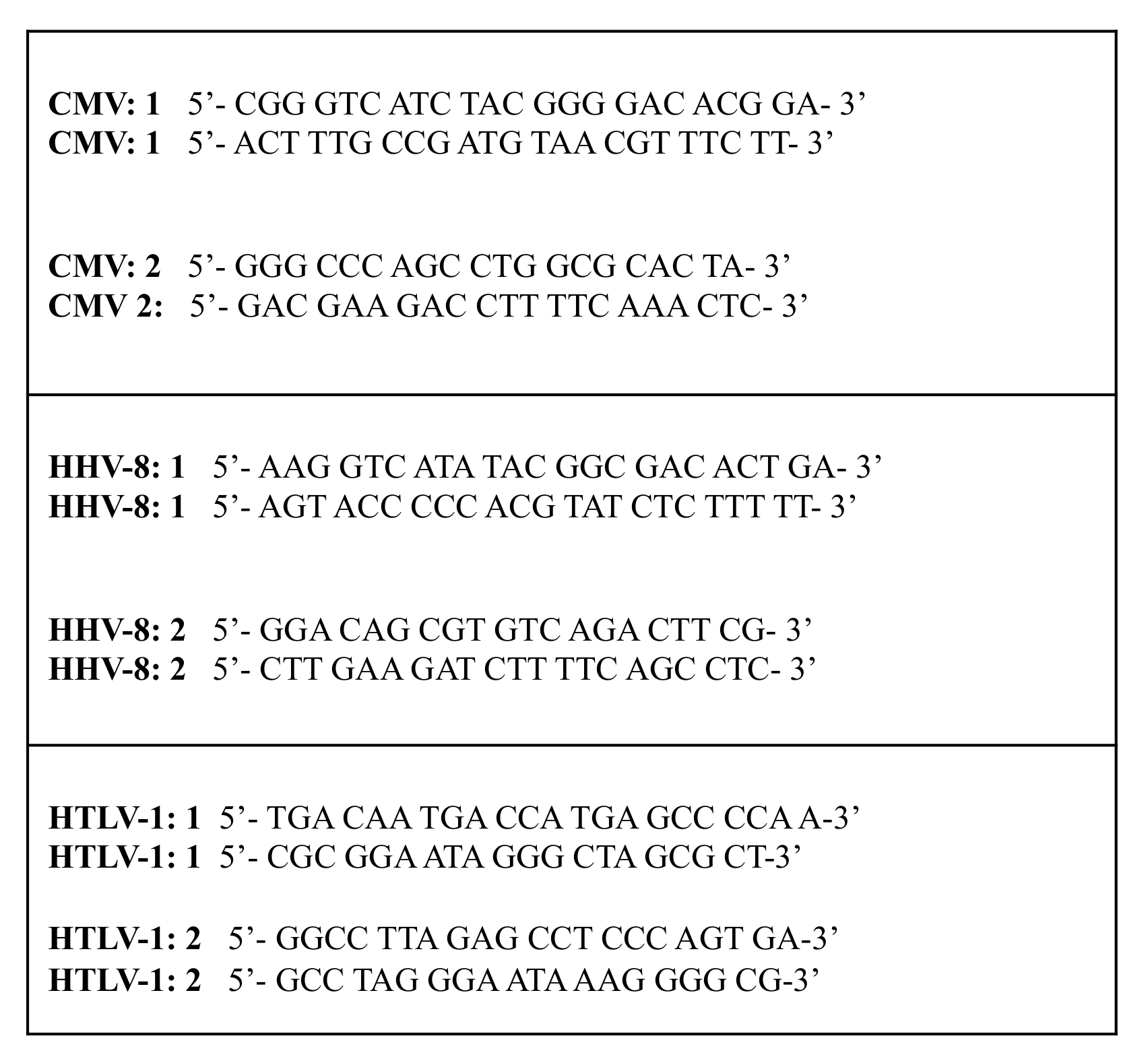

Supplement: S7 Table — (TIFF) [file ppat.1005158.s019.tiff]
