# Supplementary material for: Distinct Viral and Mutational Spectrum of Endemic Burkitt Lymphoma
Source: PLoS Pathog. 2015 Oct 15;11(10):e1005158. doi: 10.1371/journal.ppat.1005158 (PMC4607508; doi:10.1371/journal.ppat.1005158)
Supplement: S8 Table — (PDF) [file ppat.1005158.s020.pdf]

**EBNA-1 :** 5’- TAC AGG ACC TGG AAA TGG CC- 3’<br>5’- TCT TTG AGG TCC ACT GCC G-3’	**BALF-4 :** 5’-AAC CTT TGA CTC GAC CAT CG-3’<br>5’-ACC TGC TCT TCG ATG CAC TT-3’
**EBNA-2 :** 5’-TAA CCA CCC AGC GCC AAT C-3’<br>5’- GTA GGC ATG ATG GCG GCA G 5’	**BILF-1 :** 5’-GTC ACC TTC ACC GGA CTC AT-3’<br>5’-GTA GTA GCG GGC AAC GAG AG-3’
**EBNA-3c :** 5’- CTG GCA AAA CTT GCT CCA- 3’<br>5’- GTG CTT CTG CCT TAT CAG A- 3’	**BHRF-1 :** 5’-AGA AAC ACC TCT CCG CCT TT-3’<br>5’- ATC CAC ATG TTC GGT GTG TG-3’
**LMP-1 :** 5’- CAG TCA GGC AAG CCT ATG A-3’<br>5’- CTG GTT CCG GTG GAG ATG A-3’	**BMRF-1:** 5’- CAACACCGCACTGGAGAG- 3’<br>5’- GCCTGCTTCACTTTCTTGG- 3’
**LMP- 2A:** 5’- AGC TGT AAC TGT GGT TTC CAT GAC-3’<br>5’- GCC CCC TGG CGA AGA G-3’	**BLLF-1 :** ATCCAGTTGTATTCAAGGTAGG<br>ACTCATTATCACACGAACGG
**BZLF-1 :** 5’- AAA TTT AAG AGA TCC TCG TGT AAAACA TC-3’<br>5’- CGC CTC CTG TTG AAG CAG AT-3’	**LF-2 :** 5’-CTG ACC AGG ACA TCG TGC TA-3’<br>5’-GGG GTT CTT GAC CAA TCT GA-3’
**BALF-2 :** 5’-TGC ACC TGC TAG AGA ACT CG-3’<br>5’-CAC AGA GTA CGC GAC TGA GG-3’	**HPRT :** 5’-AGC CAG ACT TTG TTG GAT TTG-3’<br>5’-TTT ACT GGC GAT GTC AAT AAG-3’
